# A gut commensal bacterium promotes black soldier fly larval growth and development partly via modulation of intestinal protein metabolism

**DOI:** 10.1128/mbio.01174-23

**Published:** 2023-09-14

**Authors:** Xingyu Luo, Gangqi Fang, Kuangqin Chen, Yu Song, Tianyi Lu, Jeffery K. Tomberlin, Shuai Zhan, Yongping Huang

**Affiliations:** 1 Key Laboratory of Insect Developmental and Evolutionary Biology, Center for Excellence in Molecular Plant Sciences, Shanghai Institute of Plant Physiology and Ecology, Chinese Academy of Sciences, Shanghai, China; 2 University of Chinese Academy of Sciences, Beijing, China; 3 Department of Entomology, Texas A&M University, College Station, Texas, USA; 4 School of Environmental Science and Engineering, Shanghai Jiao Tong University, Shanghai, China; University of Hawaii at Manoa, Honolulu, Hawaii, USA; University of Texas at Austin, Austin, Texas, USA

**Keywords:** *Hermetia illucens*, *Citrobacter amalonaticus*, gut symbiont, host-microbe interaction, symbiont-mediated RNAi, intestinal protein metabolism

## Abstract

Black soldier fly (BSF), *Hermetia illucens* (L.) (Diptera: Stratiomyidae), is a promising bio-agent to transform organic wastes into valuable insect biomass, and the intestinal microbes play an essential role in this process. Here, we report that *Citrobacter amalonaticus*, a Gram-negative gut commensal bacterium of BSF larvae, can colonize the whole intestines of the larva and significantly promote the larval growth and development. The bacterial association with the larva had a marked impact on the larval transcriptome and modulated the expression of genes involved in dozens of biological pathways including host macro-nutrient metabolism and immune response. We investigated the underlying mechanisms driving the interaction and found that *C. amalonaticus* association can directly promote the expression of two gene families related to intestinal protein metabolism: Hitryp serine protease trypsin family and Himtp metallopeptidase family. To determine the gene function, we developed a symbiont-mediated double-strand RNA interference approach and successfully achieved gene knockdown in larval midgut *in situ*. Knockdown of the two gene families and protease inhibition in larval intestines attenuated the promoting effects on larval growth significantly. The results indicate that the gut symbiont promotes BSF larval growth partly via modulating the expression of the genes involved in intestinal protein metabolism. Taken together, this study establishes a novel genetic tool to study the insect functional genomics and the host-symbiont interaction and sheds light on the important roles of intestinal protein metabolism on the interaction.

**Importance:**

Black solider fly larvae and the gut microbiota can recycle nutrients from various organic wastes into valuable insect biomass. We found that *Citrobacter amalonaticus*, a gut commensal bacterium of the insect, exerts beneficial effects on larval growth and development and that the expression of many metabolic larval genes was significantly impacted by the symbiont. To identify the larval genes involved in the host-symbiont interaction, we engineered the symbiont to produce double-strand RNA and enabled the strain to silence host genes in the larval gut environment where the interaction takes place. With this approach, we confirmed that two intestinal protease families are involved in the interaction and provided further evidence that intestinal protein metabolism plays a role in the interaction. This work expands the genetic toolkits available to study the insect functional genomics and host-symbiont interaction and provide the prospective for the future application of gut microbiota on the large-scale bioconversion.

## INTRODUCTION

Black soldier fly larvae (BSFL) can transform organic wastes into valuable insect biomass and the bioconversion of organic wastes by BSFL is carbon friendly and economically sustainable and has drawn considerable attentions in the recent years (1, 2). The BSFL gut microbiota play an indispensable role in the bioconversion process (3, 4). The gut microbiota can encode enzymes such as proteases, cellulases, and lipases to facilitate the degradation of the organic wastes (3, 5, 6). The gut microbiota are also involved in degrading xenobiotics such as antibiotics and mycotoxin (7, 8). Despite recent progress on our understanding of the roles of gut microbiota on the bioconversion, the molecular mechanisms through which the gut microbes exert their beneﬁcial inﬂuences on BSFL biology are still largely unexplored.

The promoting effects of gut symbionts on larval growth and development and the underlying mechanisms have been reported in the model of the interaction between *Drosophila melanogaster* and its gut symbionts (9 – 12). The gut symbiont *Acetobacter pomorum* can regulate the growth and development, energy metabolism, and intestinal stem cell activity of *Drosophila* by regulating insulin-like growth factor signaling (12) and can also promote larval growth by synthesizing vitamin B1 (9). Another intestinal commensal bacterium, *Lactoplantibacillus plantarum*, can improve the larval sensitivity to amino acids through the target of rapamycin signaling pathway to regulate the insulin hormone signal and promote larval growth and development (11). Besides, the gut symbiont can also influence larval growth via the modulation of larval nutrient digestion and absorption. The inoculation of the fly diet with the symbiont *L. plantarum* up-regulates the expression of intestinal protease genes in gnotobiotic *Drosophila* larvae, thus increasing the activity of protease in intestine (10, 13). In the case of BSFL, Yu et al. (14) found that the existence of larval microbiota in gnotobiotic BSFL can enhance the activity of trypsin in intestines (14). However, such studies do not exclude the possible influences of the substrates that are constantly fermented by the gut bacteria on the intestinal protease activity, considering the existence of those bacteria in the substrate insect ingested (15, 16).

*Citrobacter* is a genus of Gram-negative bacteria of the family *Enterobacteriaceae* and is commonly found in the guts of various insects, such as termites, oriental fruit fly, and Colorado potato beetle (17 – 19). Bacteria in the genus are also found in BSFL intestines by 16S rRNA gene sequencing analysis (4, 8). One strain of a *Citrobacter* species that was isolated from BSFL intestine promotes BSFL growth (4). However, the molecular mechanisms of the insect-microbe interaction remain largely unknown partially due to the lack of genetic tools on those nonmodel insects.

RNA interference (RNAi) is a highly conserved mechanism against viral infection among eukaryotes and has been exploited to be both a genetic tool to study gene function in many organisms and a technology to control insect pests (20, 21). Common ways to deliver double-strand RNA (dsRNA) to insect tissues or cells include injection and feeding purified dsRNA or bacteria producing dsRNA (22). Recently, a novel dsRNA delivery strategy has emerged where the insect gut symbiont was engineered to produce dsRNA and result in RNAi effects in insects *in situ* (23 – 25). The engineered intestinal symbiont *Snodgrassella alvi* can colonize bees and constitutively produce double-stranded RNA to activate RNAi in the host, thus altering bee physiology and growth (25). Such method provides not only a novel tool to study insect functional genomics and the host-symbiont interaction but also a potential strategy of exploiting gut microbes to improve insect farming.

Modulation of the microbiota associated with BSFL has the potential to accelerate larval growth and shorten the bioconversion process (3, 4), which has great industrial value. However, the lack of understanding on the host-microbe interaction and the limited genetic tools available have stemmed further modulating microbiota in BSFL-mediated bioconversion. Here, we isolated a commensal bacterium of the BSFL *Citrobacter amalonaticus* and developed *C. amalonaticus*-mediated RNAi of host genes. We further confirmed that two intestinal protease families and the intestinal protein metabolism play important roles in the growth-promoting effects by the symbiont. The symbiont-mediated RNAi in BSFL can be further used to study the insect functional genomics and the host-symbiont interaction and tailored to improve industrial value of the bioconversion.

## RESULTS

### *C. amalonaticus* strain promotes larval growth and development

**Fig 1:**
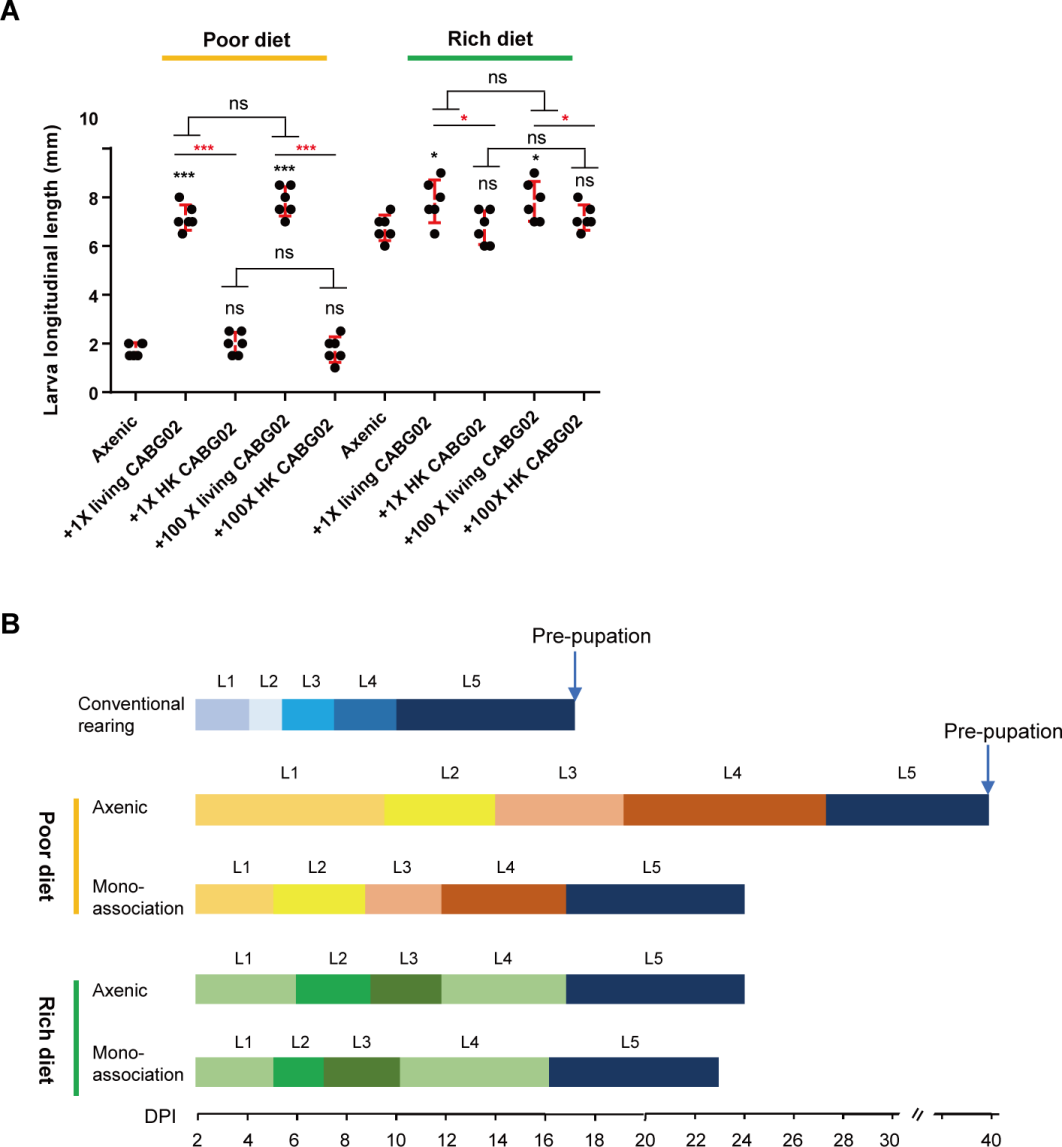
*C. amalonaticus* promotes larval growth and development. (**A**) Larval longitudinal length at 6 dpi after the association of CABG02 of 1× living bacteria, 1× heat-killed bacteria, 100× living bacteria, 100× heat-killed bacteria in both poor diet and rich diet with germ-free larvae. The control group consisted of the germ-free larvae with no bacterial association. (**B**) The duration of larval instar stages after 1× CABG02 association with sterile larvae in both poor diet and rich diet. Each treatment was replicated six times. The error bar represents mean ± SD. Asterisks just above the dot plots represent statistical significance compared to the axenic group. Red asterisks above the horizontal bars represent statistical significance between the groups associated with heat-killed bacteria and living bacteria at the same dosage applied. Black asterisks above the horizontal bars represent statistical significance between the groups associated with 1× and 100× bacteria with the same cell viability. The statistical significance was determined by one-way analysis of variance (ANOVA) and Tukey’s multiple comparison test with ^*^ *P* < 0.05, ^**^ *P* < 0.01, ^***^ *P* < 0.001; ns, not significant (*P* > 0.05).

A list of bacterial strains was isolated from BSFL intestines (Table S1), and we mono-associated the isolates with germ-free larvae in both sterile rich and poor diets to test the influence of those bacteria on larva growth. Among them, association of the strain CABG02 resulted in one of the strongest growth-promoting effects in both sterile diets, and the strain was classified as *C. amalonaticus* strain based on 16S rRNA gene sequence (Fig. S1; Table S1). At 6 days post-innoculation (dpi), the longitudinal length of the larvae associated with the strain in poor diet can reach nearly four times that of the germ-free larvae (*P* < 0.01) (Fig. 1A). In addition, the bacterial association sustains optimal larval development at both diets. It took 15 d for larvae to develop from hatching to prepupation under conventional rearing, while 40 d in poor diet and 21 d in rich diet (Fig. 1B). We also found that the growth-promoting effect was independent of the loads of inoculum and CABG02 must be alive to sustain larval growth (Fig. 1A), implying that the promotion effect did not result from larval digestion of the inoculum *per se* and was influenced by the nutrient level of the diet.

The associated bacteria may ferment the wheat bran diet resulting in the metabolic changes of the substrate, thus promoting larval growth (26). To tackle this hypothesis, we firstly inoculated the strain into the sterile poor diet and rich diet, fermenting the diet for 6 d before inactivating the bacteria inside by heat. Then, the germ-free larvae were inoculated into the diets. The results showed that fermentation of the substrate by CABG02 can promote larval growth, while the growth rate was still lower than that of the bacterial association (*P* < 0.05) (Fig. S2). This result indicates that the fermentation products partially promote larval growth while other contributing factors to the growth-promoting effect exist.

### *C. amalonaticus* strain can stably colonize larval guts

**Fig 2:**
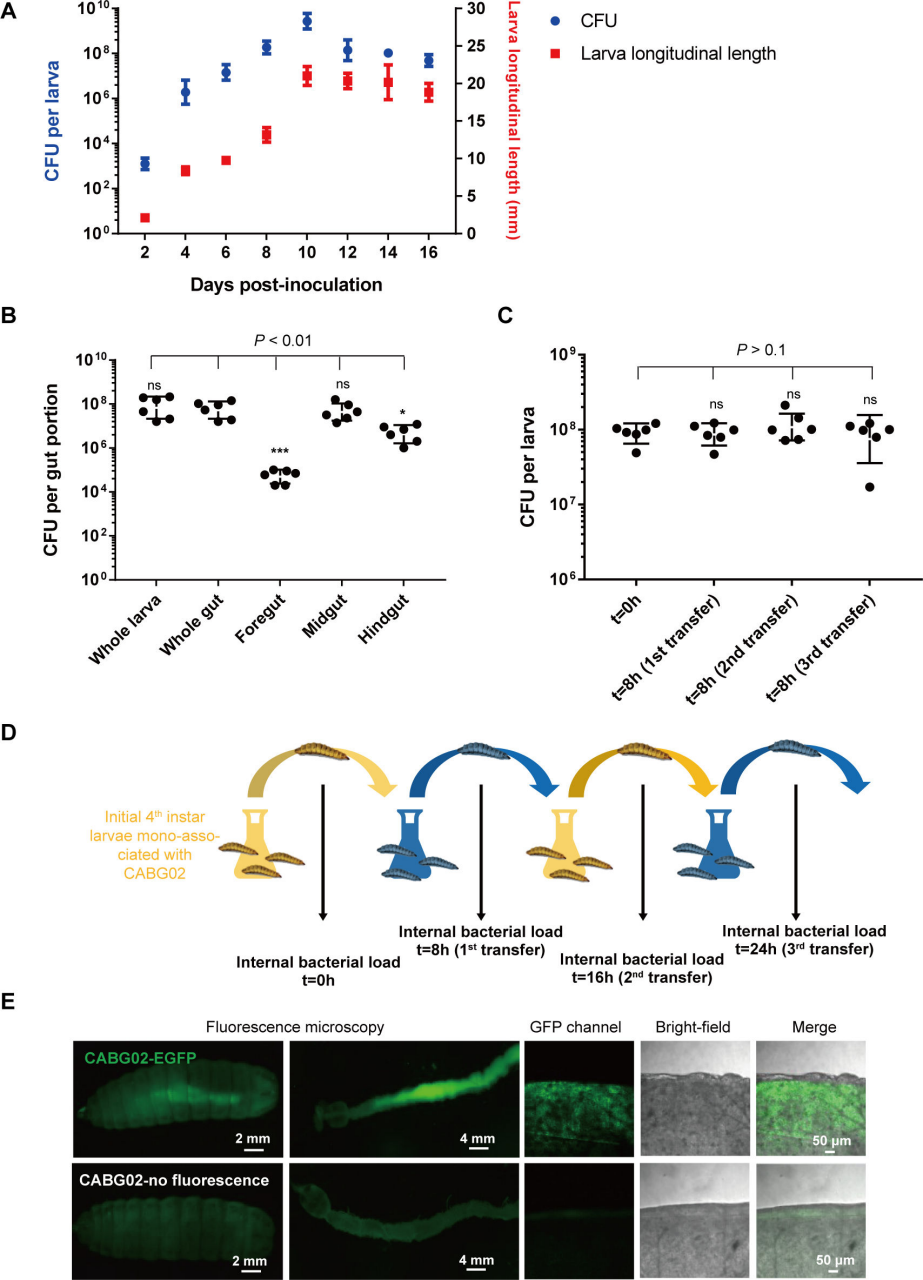
*C*. *amalonaticus* can stably colonize larval guts. (**A**) Bacterial loads of larval guts associated with 1× CABG02 at different larval developmental stages. (**B**) Distribution of the bacteria in larval gut portions of the larvae associated with 1× CABG02. (**C and D**) Bacterial loads of CABG02 in the larval guts post larval transfer (**C**) and the diagram of the experiment design (**D**). (**E**) Bacterial localization in the larvae associated with 1× nonfluorescent or fluorescent CABG02. Enhanced green fluorescent protein (EGFP) fluorescence from the engineered strain is green. Larval tissues from five individual larvae were grouped for each treatment. Each treatment was replicated six times. The error bar represents mean ± SD. Asterisks just above the dot plots in panel **B** represent statistical significance compared to the whole gut group as determined by ANOVA and Tukey’s multiple comparisons test with ^*^ *P* < 0.05, ^**^ *P* < 0.01, ^***^ *P* < 0.001; ns, not significant (*P* > 0.05).

To analyze CABG02 localization in the larval gut, we quantified CABG02’s loads in mono-associated larvae of different developmental stages and the loads in different gut sections of fourth-instar larvae. The bacterial cells were present in the larvae throughout the larval stage (Fig. 2A) and present all along the intestinal tract of fourth-instar larvae, while the midgut harbors about 10- to 100-fold more bacteria than that of hindgut and foregut, respectively (*P* < 0.05) (Fig. 2B). We next wondered if the strain persisted in the gut or transiently passed through the lumen along with the ingested food. To answer this question, we designed larval transfer experiments adapted from Storelli et al. (15) (15). After the third transfer, the number of bacteria in BSFL guts still had no significant change, indicating that the bacteria can stably colonize the intestinal tract (Fig. 2C and D). We also observed that, 12 d after colonization, the strain occupied the gut lumen and no invasion through the peritrophic membrane was observed (Fig. 2E).

### The modulation of the expression of host genes by *C. amalonaticus*

**Fig 3:**
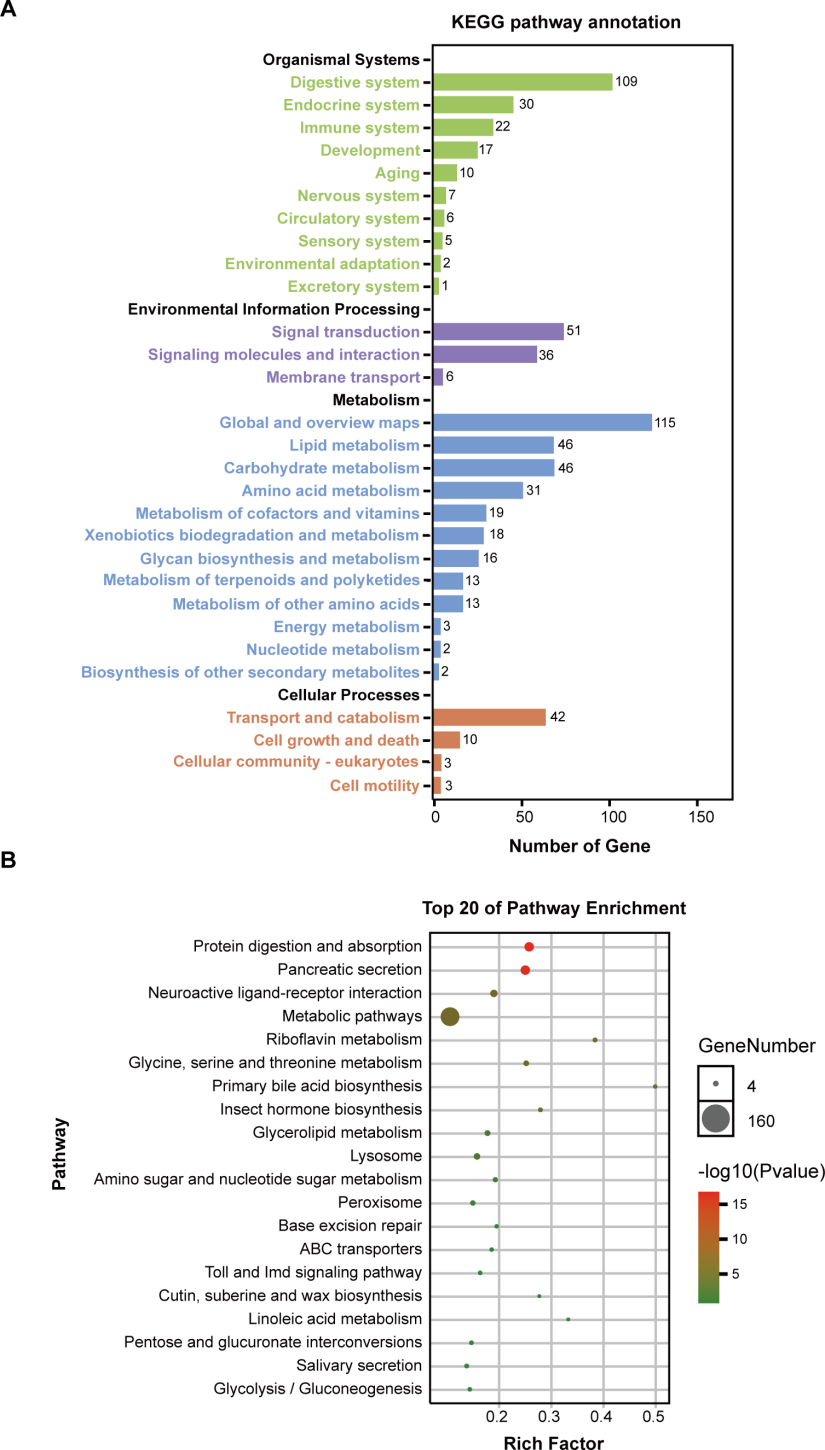
KEGG pathway enrichment of DEGs between the germ-free larvae and the larvae after 2 days of association with 1× CABG02. (**A**) The number of DEGs in each KEGG pathway. (**B**) Top 20 of the pathways enriched in KEGG analysis. Each treatment was replicated three times.

To gain insight into the molecular cross-talk between the gut symbiont and its host, we compared the transcriptomic changes between CABG02-associated larvae and germ-free larvae. After 2 d of CABG02 association, there were 1,381 genes in CABG02-associated larvae significantly differentially expressed compared to the germ-free larvae, and among those differentially express genes (DEGs), 802 genes were significantly up-regulated, and 579 genes were significantly down-regulated (Table S2). KEGG pathway analysis showed that the DEGs mainly clustered in lipid metabolism, carbohydrate metabolism, and amino acid metabolism and the pathways related to the digestive system, endocrine system, and immune system (Fig. 3A). Among them, the enrichment of protein digestion and absorption pathway and pancreatic secretion pathway is particularly significant (*P* < 0.001) (Fig. 3B). Interestingly, 12 of the DEGs were significantly enriched in the Toll and Imd signaling pathway, and the majority of them were peptidoglycan recognition proteins (*P* < 0.01) (Table S3).

### Establishment of the symbiont-mediated RNAi

**Fig 4:**
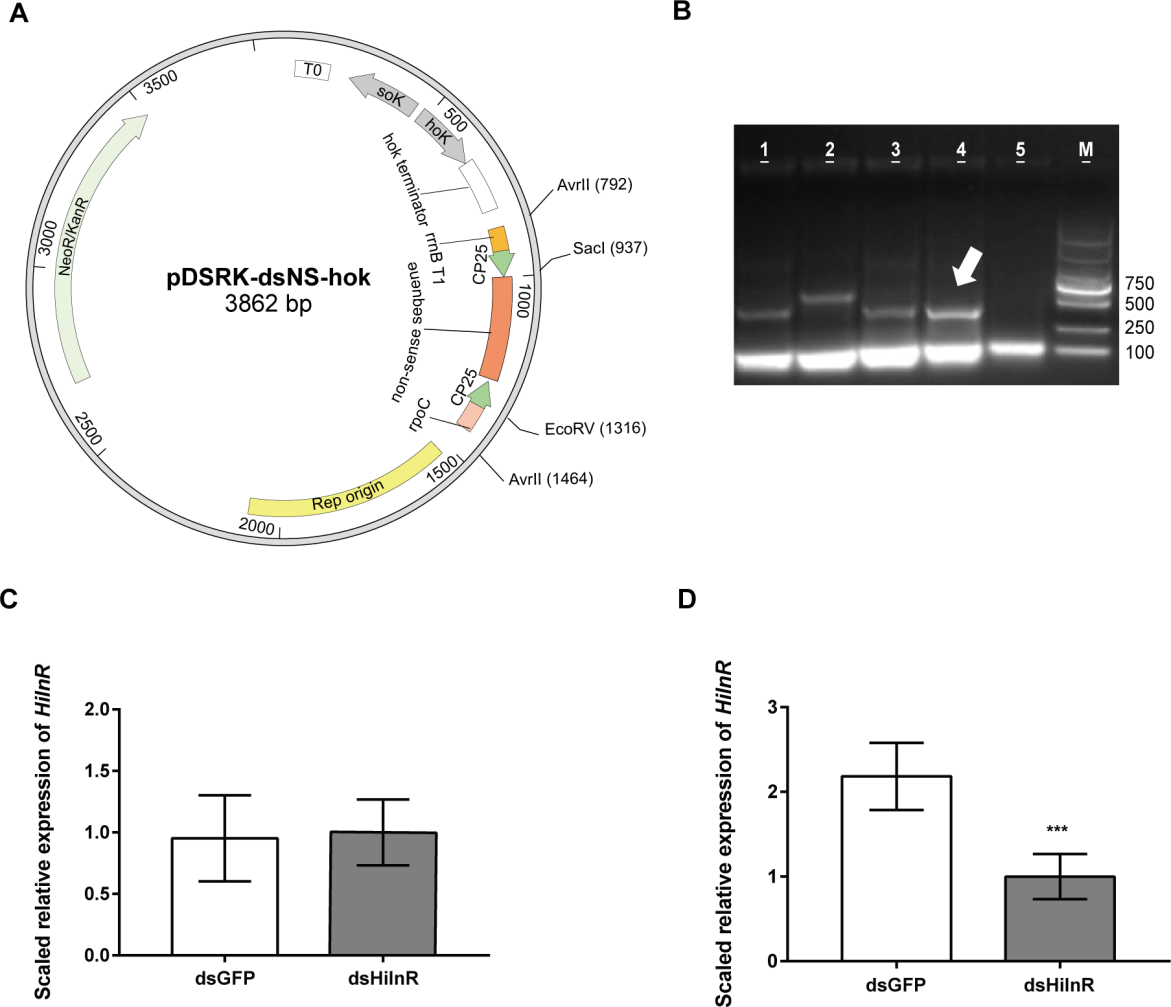
*C. amalonaticus*-mediated RNAi silences BSFL genes. (**A**) Scheme of dsRNA-producing plasmid pDSRK-dsNS-hok used in this study. (**B**) One percent agarose gel electrophoresis of crude RNA extraction samples of CABG02 Δ*rnc*::pDSRK-dsGFP-hok (lane 1), CABG02 Δ*rnc*::pDSRK-dsHiInR-hok (lane 2), CABG02 Δ*rnc*::pDSRK-dsHitryp1-hok (lane 3), CABG02 Δ*rnc*::pDSRK-dsHimtp1-hok (lane 4), and CABG02 Δ*rnc* (lane 5). The arrow indicates the band of dsRNA. (**C and D**) Real-time quantitative PCR examination on the expression of *HiInR* in the guts (**C**) and the remnant body (**D**) of the larvae that were associated with 1× CABG02 Δ*rnc*::pDSRK-dsGFP-hok (control) and 1× CABG02 Δ*rnc*::pDSRK-dsHiInR-hok at 8 dpi. Larval tissues from six individual larvae were grouped for each treatment. Each treatment was replicated four times. The error bar represents mean ± SD. Asterisks indicate statistical signiﬁcance compared to the control group as determined by a two-tailed Student’s *t*-test with ^*^ *P* < 0.05, ^**^ *P* < 0.01, and ^***^ *P* < 0.001.

To further confirm the function of the DEGs, we developed *C. amalonaticus*-mediated RNAi approach to silence the BSFL genes. First, we constructed the plasmid pDSRK-dsNS-hok that contains the elements for constitutively expressing dsRNA and the hok/sok element that can produce toxic effects on the host bacterial cell when the plasmid is lost, which, in turn, maintains the stability of the plasmid in the host in the absence of antibiotic resistance selection (27) (Fig. 4A). The functionality of the dsRNA-producing plasmid was confirmed in *Escherichia coli* HT115 strain that is defective in ribonuclease III (RNase III) and commonly used for expressing dsRNA (28) (Fig. S3). We then investigated if the dsRNA produced by *E. coli* HT115 in gut lumen can result in RNAi effects in BSFL. We selected the insulin receptor gene *HiInR* (NCBI accession ID: LOC119660051) as the target gene for the RNAi test because the gene is conserved in insects and it is expressed in the whole body of Diptera insects (29, 30). When associating the HT115::pDSRK-dsHiInR-hok with germ-free larvae, we found that the strain carrying the plasmid could significantly reduce the expression of *HiInR* only in larval intestines compared with the strain carrying pDSRK-dsGFP-hok from the control group, while the reduced expression was not observed in larval remnant body or the whole body (Fig. S4).

Next, we moved to investigate if the dsRNA-producing system can function in the gut symbiont *C. amalonaticus*. The genome of the bacterium harbors RNase III (*rnc*) gene, which may degrade dsRNA. So, we constructed the Δ*rnc* mutant strain by deleting the *rnc* gene via homologous recombination mediated by pKOV plasmid (31) (Fig. S5). The mutant strain transformed with the dsRNA-producing plasmids can constitutively produce dsRNA (Fig. 4B) and be stably harbored in the host cells in the absence of antibiotic (Fig. S3B), and it can result in RNAi effects only in the intestines of BSFL but not the remnant body similarly as observed in HT115 strain (Fig. 4C and D). Notably, the deletion of *rnc* gene did not affect the beneficial effects on larval growth and the gut colonization (Fig. S6 and S7). These results indicated that the transfer of dsRNA between cells in BSFL may be cell autonomous, and the RNAi effect cannot be delivered systematically.

### Intestinal protein metabolism is involved in the host-symbiont interaction

**Fig 5:**
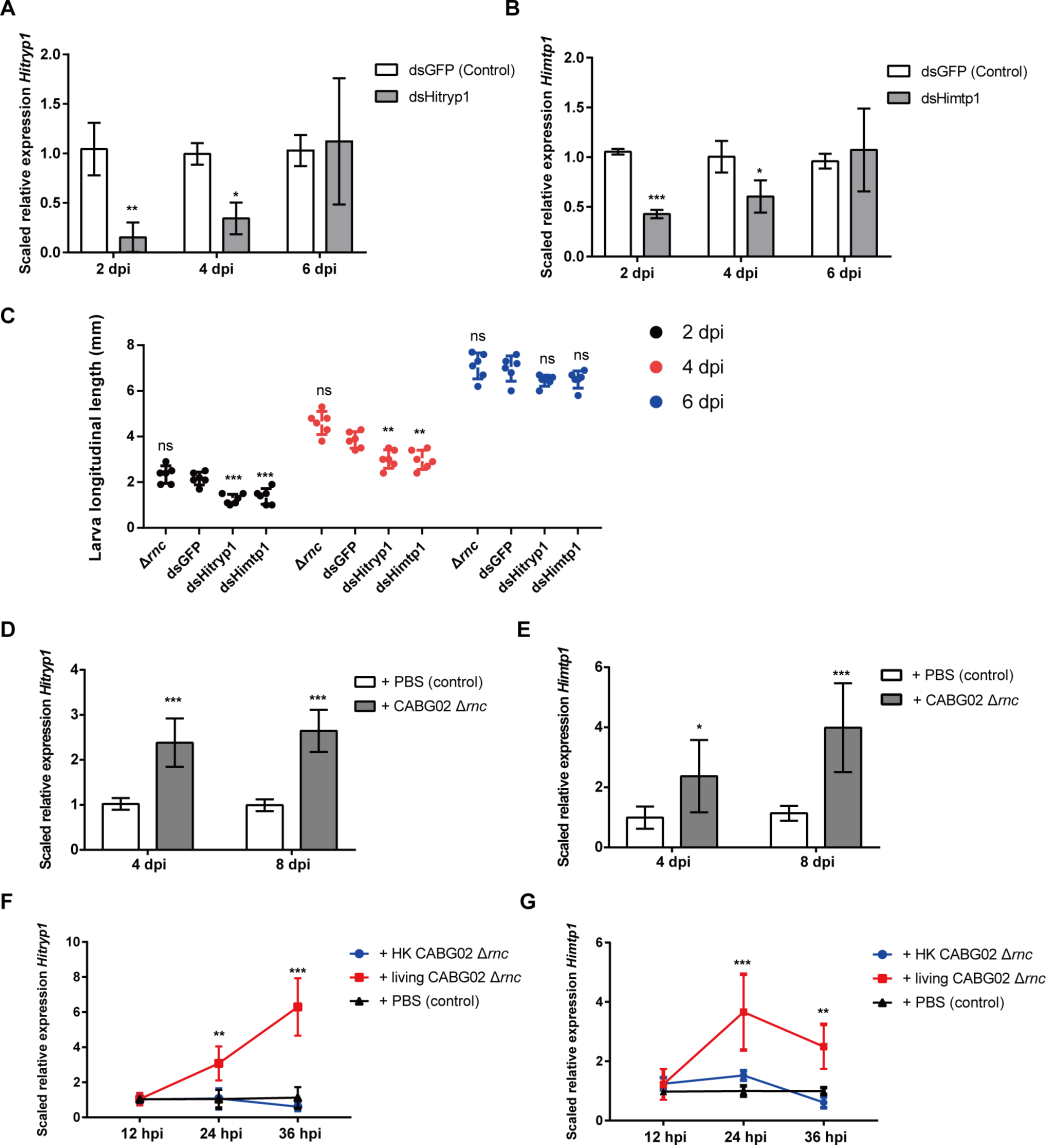
Hitryp and Himtp families are involved in the host-symbiont interaction. (**A and B**) The expressions of Hitryp (**A**) and Himtp (**B**) in the larvae that were associated with 1× CABG02 Δ*rnc*::pDSRK-dsHitryp1-hok and 1× CABG02 Δ*rnc*::pDSRK-dsHimtp1-hok, respectively, at 2, 4, and 6 dpi. The larvae associated with 1× CABG02 Δ*rnc*::pDSRK-dsGFP-hok were the control group. (**C**) Larval longitudinal length at 2, 4, and 6 dpi after the association of 1× CABG02 Δ*rnc*, 1× CABG02 Δ*rnc*::pDSRK-dsGFP-hok (control), 1× CABG02 Δ*rnc*::pDSRK-dsHitryp1-hok, 1× CABG02 Δ*rnc*::pDSRK-dsHimtp1-hok with germ-free larvae in poor diet. (**D and E**) The expressions of family Hitryp (**D**) and Himtp (**E**) in the whole body of germ-free larvae or the larvae associated with 1× CABG02 Δ*rnc*. (**F and G**) Direct up-regulation on the expressions of family Hitryp (**E**) and Himtp (**G**) in the intestines of the fourth-instar larvae after transferring to inorganic medium upon association with 1× CABG02 Δ*rnc*, 1× heat-killed CABG02 Δ*rnc,* and phosphate-buffered saline (PBS) buffer (control) for 12, 24, and 36 h. Larval tissues from six individual larvae were grouped for each treatment. Each treatment was replicated four times. The error bar represents mean ± SD. Asterisks indicate statistical signiﬁcance compared to the control group as determined by a two-tailed Student’s *t*-test (**A and B, D and G**) or one-way ANOVA and Tukey’s multiple comparison test (**C**) with ^*^ *P* < 0.05, ^**^ *P* < 0.01, and ^***^ *P* < 0.001; ns, not significant (*P* > 0.05).

With *C. amalonaticus*-mediated RNAi, we screened a subset of the DEGs that had great statistical significance and potential biological significance and were predicted to be expressed in BSFL intestines based on BSFbase (https://insectomics.net/BSFbase/) (32). The screen was conducted in sterile rich diet where larvae were associated with 1× CABG02 Δ*rnc* transformed with dsRNA-producing plasmids targeting various candidate genes, respectively. Larvae were collected at 4 dpi, and the results were shown in Fig. S8. We found that when the serine protease gene *Hitryp1* (LOC119654750) and metallopeptidase gene *Himtp1* (LOC119656089) were knocked down (Fig. 5A and B), the growth-promoting effect mediated by CABG02 strain was attenuated (Fig. 5; Fig. S8).

A multiple alignment among genes of similar coding sequence in the genome with the two protease genes revealed that several genes of great similarity in coding sequence with the two protease genes are present in BSF genome. The coding sequence of the gene copies within Hitryp family is >90% identical to each other, while >95% identical for Himtp family (Fig. S9 and S10). Due to the great homology in coding sequence, the whole gene families were further characterized instead of the individual genes within the gene families in the following study.

A further characterization of the two gene families revealed that both of them were continuously expressed in the early larval developmental stages (Fig. 5D and E) and were mainly expressed in BSFL intestines (Fig. S11). In addition, the association of *C. amalonaticus* can significantly increase the expression of the two gene families in BSFL at 4 and 8 dpi (*P* < 0.05) (Fig. 5D and E). The up-regulated expression of these two protease families observed may be due to the bacterial fermentation of the substrate wheat bran, which hydrolyzes proteins in the wheat bran and increases the number of polypeptides and amino acids directly ingested by the larvae, thus increasing the expression of intestinal protease genes (33). To answer the question, we developed a novel inorganic medium consisting of fine sand and biochar. The surface of biochar particles can attach CABG02 bacterial cells, and the particles can be ingested by larvae and the bacterial cells can enter the intestine through mouthpart alongside (34). We transferred germ-free fourth-instar larvae into the sterile inorganic medium and then associated the larvae with CABG02, the heat-killed CABG02, and PBS buffer, respectively. We found that the expression of these two gene families in larval intestines was still significantly up-regulated in larvae-associated living bacteria (Fig. 5F and G), indicating that *C. amalonaticus* can directly up-regulate Hitryp and Himtp families in larval guts independent of any nutrients in the food.

**Fig 6:**
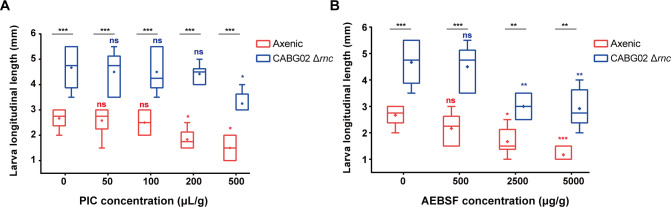
Intestinal protein metabolism is partly involved in the growth-promoting effects of the symbiont. (**A and B**) Larval longitudinal length at 6 dpi after the inoculation of 1× CABG02 Δ*rnc* in the sterile poor diet supplemented with a concentration of 0, 50 µL/g, 100 µL/g, 200 µL/g, 500 µL/g of PIC (**A**) and 0, 500 µg/g, 2500 µg/g, 5000 µg/g AEBSF (**B**). The error bar represents mean ± SD. Asterisks just above the dot plots represent statistical significance between longitudinal length of the larvae in the axenic group (red asterisks) or the CABG02 Δ*rnc* group (blue asterisks) and the length of respective larvae when no protease inhibitor was supplemented. Black asterisks above the horizontal bars represent statistical significance between axenic group and CABG02 Δ*rnc* group at a given concentration. Each treatment was replicated six times. The statistical significance was determined by two-way ANOVA and Tukey’s multiple comparison test with ^*^ *P* < 0.05, ^**^ *P* < 0.01, ^***^ *P* < 0.001; ns, not significant (*P* > 0.05).

To further confirm the roles of intestinal protein metabolism in the interaction, we added two types of protease inhibitors in the diet to investigate the effects of inhibited protease activities in larval growth. When protease inhibitors protease inhibitor cocktail (PIC) and a specific irreversible serine-protease inhibitor (AEBSF) were added to the poor diet inoculated with germ-free larvae or CABG02-associated larvae, respectively, the larval growth was significantly reduced as expected (*P* < 0.05) (Fig. 6). Interestingly, larvae were less sensitive to protease inhibitors when associated with CABG02, and when the concentration of protease inhibitors was high, the growth- promoting effect mediated by CABG02 was also significantly weakened (*P* < 0.05) but still can be clearly observed (Fig. 6). The above results suggest that protease families Hitryp and Himtp are involved in CABG02-mediated growth promotion, and intestinal protein metabolism plays at least a partial role in the host-symbiont interaction.

## DISCUSSION

A study by Yu et al. (14) has shown that inoculating gut microbiota into sterile BSFL can enhance larval protein digestion by enhancing activities of protein digestive enzymes in larval intestines, and the protease such as trypsin and peptidase plays a major role in protein degradation by BSFL (14). The observation was consistent with what we found in the case of BSFL gut symbiont *C. amalonaticus*. We found the symbiont can promote larval growth and development (Fig. 1) and modulate the expression of Hitryp and Himtp families in larval intestines independent of nutrients (Fig. 5F and G); furthermore, we determined that silencing the protease genes by symbiont-mediated RNAi or inhibition of protease activities significantly attenuated the growth-promoting effects (Fig. 5A through C, and Fig. 6).

The multiple gene copies within the two protease families may arise from gene duplication events commonly found in BSF. The duplication events of the two proteases also exist in the genome of housefly *Musca domestica*. Given that the housefly and BSF live in ecologically similar niches, the duplication of these two protease genes may facilize the recycling of various organic wastes by the two species (35). Interestingly, the members in Himtp family were all up-regulated in the first-instar larvae associated with the symbiont, while the members in Hitryp family exhibited a varying expression pattern (Table S2). Albeit great homology in biochemical function, the members in the Hitryp family seem to be subjected to a different regulation at the early larval developmental stage. The dsRNA-mediated RNAi approach adopted in this study is able to interfere the expression of all members in the two gene families, considering that the RNAi target region of the genes shares great sequence similarity with their duplicates (Fig. S9 and S10). The serine protease trypsin Hitryp may be directly involved in the hydrolysis of proteins, or it may act as an early trypsin as a signal to regulate the expression of trypsin in the later developmental stage (36). The metallopeptidase Himtp is an astacin-like protein, which may be directly involved in the hydrolysis of peptides and may also be involved in the activation of growth signals (37). Detailed function and regulation of the two protease families require further investigation.

Intriguingly, we found that the symbiont also regulates the expression of larval genes involved in immune response (Table S3). When bacteria enter the intestinal tract of insects, components of cell wall such as peptidoglycan (38) and teichoic acid (39) will be recognized by the receptors of insect intestinal epithelial cells, thus leading to the up-regulation of the immune pathway Toll or Imd pathway in insect cells (40). It can be seen from Table S3 that when the CABG02 strain was present, the expression of multiple peptidoglycan recognition protein-like genes in the larvae was significantly increased, indicating that the symbiont could be recognized by the peptidoglycan recognition protein in BSFL. The recognition by the intestinal epithelial cells should be the first step in the host-symbiont interaction; then how does it lead to the increasing expression of protease genes in the cells afterwards? Studies have proposed that, in the primitive digestive tract of early animal evolution, immunity and digestion may have a common evolutionary origin (41, 42). The complex cell signal transduction pathways behind the direct up-regulation of intestinal protease gene expression by *C. amalonaticus* in BSFL remain to be further studied.

In addition to the protein metabolism, we also found that the symbiont can influence metabolic genes related to other macronutrients, such as lipids and polysaccharides, and many genes in the digestive system pathway from transcriptomics (Fig. 3). Together with the observed impact of diet nutrient level and metabolites from the bacterial fermentation in the diet on larval growth (Fig. 1; Fig. S2), the results suggest that the beneficial effects of *C. amalonaticus* may also be influenced by larval digestion and absorption of other nutrients in the substrate.

In this study, we successfully developed the gut symbiont *C. amalonaticus*-mediated RNAi in BSFL. To our knowledge, this is the first time to deliver dsRNA via gut symbiont in Dipteran insects and to apply this approach to study the interaction between BSFL and its gut symbiont. Unfortunately, our results indicated that the RNAi effect induced by this approach cannot be delivered systematically in BSFL (Fig. 4; Fig. S4). Nonsystemic delivery of naked dsRNA does not trigger effective gene silencing in some Dipteran insects (43), and the mechanisms of dsRNA transfer among animal cells remain to be deciphered. In the case of gene families Hitryp and Himtp, the RNAi effect could be observed after 2 and 4 days of the association of dsRNA-producing strain but was not detected at 6 dpi (Fig. 5A and B). One explanation is that when the larvae entered the third instar at 6 dpi, the larval body without gut portion grew larger, and the sampling of whole body instead of the gut portion affected the real-time quantitative PCR (qRT-PCR) detection. Himtp and Hitryp families are mainly expressed in larval intestine (Fig. S8), and the mRNA of the remnant body may mask the detection of the target genes in qRT-PCRs. Another possibility is that the high doses of dsRNA reduced the efficiency of RNAi (44). The phenomenon that the RNAi of target genes causes the overexpression of the target genes exists in a variety of insects (45 – 47). In addition, the growth-promoting effect was restored at 6 dpi (Fig. 5C), coinciding with the restoration of target gene expression, suggesting the roles of the genes in larval growth and development. On the other hand, the growth restoration may result from the intrinsic property of the two gene families or the compensation for the knockdown by other proteases of similar function in the larval guts.

*C. amalonaticus* was commonly found in animal guts and plays important roles in host physiology (48, 49). For example, in mouse intestines, *C. amalonaticus* can inhibit the proliferation of the intestinal pathogen *Citrobacter rodentium* through cell-to-cell contact and provide colonization resistance for the antibiotic-treated mouse intestines (48). A strain of *C. amalonaticus* was also screened in the human gut and determined to be capable of metabolizing choline and producing acetate (49). *C. amalonaticus* mutant with RNase III deficiency developed here can constitutively express dsRNA if transformed with the dsRNA-producing plasmid that we constructed. Considering that a variety of animals such as shrimps, insects, and planarians can trigger RNAi effects by feeding exogenous dsRNA (43, 50 – 52), this strain has the potential to be used to produce dsRNA and result in RNAi in animals of different species.

Several studies have shown that the BSFL gut microbiota play important roles in BSFL-mediated bioconversion and larval growth and development (3, 4, 8), but the molecular mechanisms underlying remain largely unelucidated. Given the importance of gut microbiota during BSFL-mediated bioconversion, our work, which characterized the roles of intestinal protein metabolism underlying the host-symbiont interaction and engineered the symbiont to modulate BSFL physiology, expands the genetic toolkits to study BSF functional genomics and host-microbe interaction and provides the prospective for the future application of gut microbiota on BSFL-mediated bioconversion.

## MATERIALS AND METHODS

### Insect rearing, diets, and chemicals

BSFL were obtained from a laboratory-maintained colony originally sampled in Wuhan, China (35). The newly hatched larvae were reared in moistened wheat bran (1:2 ratio of dry matter/ddH_2_O). For generating germ-free larvae, the egg surface was sterilized as described by Gold et al. (53). The sterilized eggs were placed into the sterile 50-mL centrifuge tubes with sterile wheat bran. The tubes were covered with sterile aluminum foil to maintain sterility and air availability and maintained on a clean bench. Sterility was checked before each sample collection by plating and 16S rRNA gene PCR. The larval rearing was carried out at 27°C with 70% relative humidity.

Rich diet was obtained by adding 5% (wt/wt) yeast exact (Catalog #: LP0021B, Oxoid) and 10%(wt/wt) tryptone (Catalog #: CM0087B, Thermo Scientific) into the sterile wheat bran. In addition, poor diet was solely wheat bran. Blue diet is the rich diet supplemented with 1% (wt/wt) erioglaucine disodium salt powder (Product #: 861146, Sigma-Aldrich). All the diets used in the study have a 1:2 ratio of dry matter/ddH_2_O and were contained in 50-mL centrifuge tubes. The inorganic medium used in the study was obtained by adding 20 g fine sand (diameter ranging from 0.10 to 0.25 mm) and 1% (wt/wt) biochar and 5-mL ddH_2_O in 50-mL sterile centrifuge tubes. Specific irreversible serine-protease inhibitor (AEBSF) was purchased from Beyotime (Catalog #: SG2000). PIC was purchased from Cell Signaling Technology (Product #: 5871). Wheat bran was purchased from a local farmer market.

### Bacterial isolation and bacterial association with larvae

The bacterial strains were isolated from the intestines of BSFL reared in the laboratory on Luria-Bertani (LB) and deMan Rogosa Sharpe (MRS) media. The isolates were identiﬁed by sequencing of 16S rRNA gene with primers 27F/1,492R (54). Before the inoculation, the bacteria were cultivated to OD_600_ = 0.5 in LB broth medium at 37°C with shaking at 220 rpm, and 1 mL of the culture was centrifuged at 4,000 rpm for 2 min to collect bacterial pellets. Then, the pellet was washed with 1× PBS buffer for three times and suspended in 500-µL 1× PBS to make a “1×” inoculum. The “100×” inoculum consisted of bacterial pellet collected from 100 mL of the culture. Then, all of the bacteria collected are inoculated onto 5 g of the sterile or unsterile diet where about 20 sterile eggs were seeded. “1×” inoculum was about 8 × 10^8^ colony formation units (CFUs) of the bacteria. For the controls, an equal volume of sterile 1× PBS was inoculated. For heat-inactive bacteria, the inoculum was prepared by placing the bacterial culture in an oven at 65°C for 4 h. The chemicals were included in the diets at indicated concentration. Six biological replicates were set up for each treatment.

### Measurement on larval growth and development

For measuring larval longitudinal length, the larvae were placed on ice for 3 min before measuring, and the average length of six larvae was recorded for each treatment. Six biological replicates were set up for each treatment. For measuring the larval development, the time it takes for the first larvae of a treatment group to reach the next instar stage was recorded as the time period required to grow to the corresponding instar stage. Three biological replicates were set up for each treatment.

### Bacterial load quantification

Bacterial loads were quantified by plating serial dilutions of lysates of larvae or gut sections associated with CABG02 on LB plates (five larvae were included for each treatment). All of the larvae subjected to bacterial load quantification were reared in sterile rich diet. Six replicates were set up for each experimental condition. The larvae or the gut sections were dissected aseptically into a 1.5-mL Eppendorf tube (EP) tube containing glass beads and 500-µL PBS buffer. Then, the samples were homogenized. The lysates were serial diluted and then spread on LB plates. The CFUs growing on the plates were counted, and the total number of bacteria in the sample was calculated according to the dilution factor.

### Germ-free larva transfer

For the colonization test and the test in inorganic medium, BSFL associated with the bacterial strain were grown in rich diet for 10 days (fourth-instar larvae), and 20 larvae were aseptically transferred to the respective diets. The detailed methods and procedures were described in Text S1.

### Plasmid construction

A 432-bp fragment containing the dsRNA-producing cassette was *de novo* synthesized by Shanghai Sunny Biotech Co., Ltd. This cassette contained a pair of bidirectional synthetic constitutive promoter CP25, two transcription terminators rrnB T1 and rpoC, and a 385-bp nonsense sequence which was inserted between the two converging promoters. The aim of inserting a nonsense sequence was to prevent the occurrence of hairpin structure and facilitate sequencing reaction. Both ends of the nonsense sequence contained a restriction site (SacI and EcoRV, respectively), which can be cut by enzymes and replaced with target dsRNA sequence. The AvrII restriction sites were designed on both sides of the cassette to facilitate the replacement of the plasmid backbone vector. The cassette was then used to replace the sequence between the NdeI and BspQI restriction sites of pUC57 plasmid, thus yielding pDSR-dsNS. A 1.0-kb fragment containing the kanamycin-resistant gene was amplified from pBBR1MCS-2 (55) by primers pUCKan F/R. The resulting fragment was used to replace the sequence between VspI and subsp. site of pDSR-dsNS. The resulting kanamycin-resistant plasmid was named pDSRK-dsNS. A 673-bp fragment containing the hok/sok element was amplified from pMATING2α (27) by using primers pMThoksok F/R. pDSRK-dsNS was reversely amplified to linearization with primers pDSRKREV F/R, and the hok/sok element was recombined into pDSRK-dsNS to obtain plasmid pDSRK-dsNS-hok.

The construction of a series of dsRNA-producing plasmids, EGFP-expressing plasmid, and *rnc*-deletion plasmid were summarized in Text S1. All primers used in this study were listed in Table S4. The sequence of plasmids generated was listed in Text S2.

### RNA extraction and cDNA synthesis

Total mRNA of various larval tissues and dsRNA of bacterial strains were both isolated using the TRIzol reagent (Invitrogen) according to the manufacturer’s instructions. The samples of mRNA were treated with DNase I (Invitrogen) to eliminate genomic DNA, and cDNA was synthesized using the ReverAid First Strand cDNA Synthesis Kit (Thermo Fisher Scientific). The crude dsRNA extraction samples were analyzed by 1% agarose gel electrophoresis to check the dsRNA band of expected size.

### Real-time quantitative PCR

Real-time quantitative PCR was performed using SYBR green (TaKaRa) to examine the expression of *HiInR*, Hitryp family, and Himtp family in larval tissues and *rnc* gene in CABG02 strain with primers HiInR_qRT-PCR F/R, Hitryp1_qRT-PCR F/R, Himtp1_qRT-PCR F/R, and rnc_qRT-PCR F/R, respectively. The detailed methods and procedures were described in Text S1.

### RNA-seq analysis

Total mRNA was collected from the whole body of 10 individual larvae associated with 1× CABG02 and 10 individual larvae without bacterial association (control) in sterile rich diet at 2 dpi. Three biological replicates were set up for each treatment. The libraries were constructed according to the manufacturer’s instructions using the TruSeq Stranded mRNA LT Sample Prep Kit (Illumina). The detailed methods and procedures were described in Text S1. The RNA-seq analysis data were included in Table S2.

### Construction of dsRNA-producing strain

The construction of the recombinant CABG02 Δ*rnc* strain was summarized in Text S1. The mutant was named as CABG02 Δ*rnc*. A series of dsRNA-producing plasmids targeting various BSFL genes were transformed into the mutant respectively by electroporation, and the growing colonies were selected from LB plates containing kanamycin (100 µM) maintained at 37°C overnight. The ability of the transformants to produce dsRNA was verified by 1% agarose gel electrophoresis. The dsRNA targeting region of the tested genes was summarized in Text S3.

### Statistical analysis

Analysis of the statistical significance of the data was performed using GraphPad Prism version 7. One-way ANOVA with Tukey’s multiple comparison test was used to test the statistical significance of differences among different treatments.

## Data Availability

All data generated or analyzed were included in the article and the supplemental material. All raw sequencing reads of the RNA-seq analysis were deposited into NCBI SRA (accession number: PRJNA973253).

## SUPPLEMENTAL MATERIAL

Text S1 mbio.01174-23-s0001.docx Additional experimental details.

Text S2 mbio.01174-23-s0002.docx Sequence and annotation of plasmids generated in the study.

Text S3 mbio.01174-23-s0003.docx dsRNA target region of the genes screened in the study.

Supplemental figures mbio.01174-23-s0004.docx Figures S1 to S11.

Legends mbio.01174-23-s0005.docx Legends to the supplemental tables and texts.

Table S1 mbio.01174-23-s0006.xlsx Information on the intestinal isolates.

Table S2 mbio.01174-23-s0007.xlsx Data of the RNA-seq analysis.

Table S3 mbio.01174-23-s0008.docx DEGs in the Toll and Imd signaling pathway from the RNA-seq analysis.

Table S4 mbio.01174-23-s0009.docx The primers used in the study.

ASM does not own the copyrights to Supplemental Material that may be linked to, or accessed through, an article. The authors have granted ASM a non-exclusive, world-wide license to publish the Supplemental Material files. Please contact the corresponding author directly for reuse.
