# Supplementary material for: A gut commensal bacterium promotes black soldier fly larval growth and development partly via modulation of intestinal protein metabolism
Source: mBio. 2023 Sep 14;14(5):e01174-23. doi: 10.1128/mbio.01174-23 (PMC10653789; doi:10.1128/mbio.01174-23)
Supplement: Text S1 — Additional experimental details. [file mbio.01174-23-s0001.docx]

**Text S1** Supplemental methods.

**The colonization test by larva transfer**

BSFL associated with the bacterial strain were grown in rich diet for 10 days (4^th^ instar larvae), and 20 larvae were aseptically transferred to sterile blue diet. After 8 hours of feeding, the gut content prior to transfer was completely expelled (blue dye can be seen throughout the larval guts), at which time the larvae are removed and surface sterilized with 75% alcohol, and then transferred to a non-stained sterile rich diet aseptically to finish the second transfer. After 8 hours, the larvae were taken out again, and the surface was sterilized and then transferred to a sterile blue diet again to complete the third transfer. Before each transfer, 5 larvae were taken to analyze the colonization of the strain. The experiment was conducted in six biological replicates.

**The gene expression test in inorganic medium**

BSFL associated with the bacterial strain were grown in rich diet for 10 days (4^th^ instar larvae), and 20 larvae were aseptically transferred to inorganic medium which was inoculated with 1X CABG02 strain, 1X heat-killed CABG02 strain, and PBS buffer respectively aseptically. After 12, 24, and 36 hours, 6 larvae were taken out from each tube and the intestines were dissected for analysis of gene expression. Four biological replicates were set up for each treatment.

**Construction of a series of dsRNA-expressing plasmids**

With pBac-GFP-Cas9 (1) as a template and pdsGFP F/R as primers, a 372 bp dsGFP fragment was amplified. With the BSFL intestinal cDNA as a template, pdsHiInR F/R, pdsHitrp1 F/R, pdsHimtp1 F/R, pdsXM_038055439.1 F/R, pdsXM_038069227.1 F/R, pdsXM_038052062.1 F/R, pdsXM_038059243.1 F/R, pdsXM_038060271.1 F/R and pdsXM_038063869.1 F/R were used as primers to amplify the dsRNA fragments dsHiInR (584 bp), dsHitrp1 (314 bp), dsHimtp1 (370 bp), dsXM_038055439.1 (345 bp), dsXM_038069227.1 (321 bp), dsXM_038052062.1 (346 bp), dsXM_038059243.1 (410 bp), dsXM_038060271.1 (389 bp), dsXM_038063869.1 (402 bp) targeting the respective genes. The plasmid pDSRK-dsNS-hok was double digested with SacI and EcoRV enzymes, and the obtained linear plasmid fragment was subjected to homologous recombination with the above-mentioned amplified dsRNA fragment to obtain the recombinant plasmid pDSRK-dsGFP-hok, pDSRK-dsHiInR-hok, pDSRK-dsHitrp1-hok, pDSRK-dsHimtp1-hok. The plasmid pDSRK-dsGFP-hok were transformed into *E. coli* HT115 strain and CABG02 Δ*rnc* strain to confirm the functionality of the plasmid. The stability of the plasmid in both strains was checked as described by Lenski and Bouma (1987) (2). Briefly, bacterial populations were cultured continuously in LB broth medium without antibiotic selection. The culture generation time was set as 4 hours. Samples were plated every five generation on antibiotic-free LB plates and 100 colonies from each sample were tested on LB plates with kanamycin (100 μM) to check the presence of the plasmid. Three biological replicates were set up for each treatment.

**Construction of EGFP-expressing plasmid**

A 785 bp fragment containing ribosomal binding site and EGFP coding sequence was amplified from pBac-GFP-Cas9 by using primers EGFP_cp25 F/R. The resulting fragment was used to replace the sequence between SacI and EcoRV site of pDSRK-dsNS-hok, yielding recombinant plasmid pDSRK-EGFP-hok. An 845 bp fragment containing CP25 promoter, ribosomal binding site and EGFP coding sequence was amplified from pDSRK-EGFP-hok by using primers pBBR_cp25 F/R. The resulting fragment was used to replace the sequence between PvuI and Mph1103I site of pBBR1MCS-2, yielding pBBR1MCS-2-cp25EGFP which can constitutively express EGFP driven by CP25 promoter.

**Construction of** ***rnc*-deletion plasmid**

pKOV plasmid was adopted as vector to delete *rnc* gene in *C. amalonaticus* genome. The strain isolated is resistant to chloramphenicol. The resistance marker of pKOV was then replaced with kanamycin resistance: The pKOV was cut by Esp3I enzymes the fragment amplified by pKOVKan F/R from pBBR1MCS-2 conferring kanamycin resistance was cloned into the linearized plasmid, yielding pKOVK. Approximately 500 bp homologous regions upstream and downstream of *rnc* gene was amplified from *C. amalonaticus* genomic DNA with primers pKOV_KO_rnc_up F/R and pKOV_KO_rnc_down F/R respectively. The two fragments were fused by overlap extension PCR, devoid of the *rnc* gene. The resulting fragment were cloned into the BamH1 and Not1 restriction site of the pKOVK vector, yielding pKOVK-Δrnc.

**Construction of** Δ***rnc* mutant**

The procedures to deletion genes in *C. amalonaticus* was adapted from Ainala et al. (2017) (3). pKOVK-Δrnc was transformed into the recipient strain CABG02 by electroporation and the growing colonies were selected from LB plates containing kanamycin (50 μM) maintained at 30°C overnight. Those colonies were then plated on the same medium but kept at 43°C overnight. The positive integrant colonies growing from the plate were then cured of the plasmid by plating on 8% sucrose medium at 30°C overnight. The sucrose-resistant and kanamycin-sensitive colonies were screened by PCR with primers pKOVKOrnc_confirm F/R, and sequencing for the desired gene replacement. The expression of *rnc* on the positive mutants was then checked by qRT-PCR.

**Fluorescent microscopy**

1X CABG02::pBBR1MCS-2 (control) and 1X CABG02::pBBR1MCS-2-cp25EGFP were associated with newly hatched germ-free larvae in the rich diet respectively. At 12 dpi, we dissected the intestines of the insect and observed the larval body and the intestine under a fluorescent microscope (Nikon AZ100). The dissected intestines were mounted on a glass slide and imaged with a Zeiss 710 confocal laser scanning microscope (20x objective). The images were analyzed with Imaris software (<https://imaris.oxinst.com>).

**qRT-PCR analysis**

The housekeeping gene *actin* and the 16s RNA gene were used as internal references with primers Hi_actin F/R (4) and 16S_qRT-PCR F/R (5), respectively. The 2^−∆∆Ct^ method (6) of relative quantification was used to examine the qRT-PCR results. Larval tissues from six individual larvae were grouped for each treatment. Samples were analyzed in 4 biological replicates. Real-time quantitative PCR (qRT-PCR) was conducted by Applied Biosystems StepOnePlusTM real-time PCR detection system and the detailed information is shown as follows:

| The reaction mixture (20 μL) | | |
| --- | --- | --- |
| SYBR green Master Mix | 10 μL | TaKaRa, Japan |
| forward primer | 0.5 μL |  |
| reverse primer | 0.5 μL |  |
| DNA template | 2 μL | 10-fold dilutions of extracted DNA sample |
| dd H2O | 7 μL | DNase free water |
| The PCR conditions | | |
| Denaturing | 95 ºC | 2:00 min |
| 39 cycles | 95 ºC | 0:15 min |
|  | 53.0 ºC | 0:30 min |
|  | 72 ºC | 0:15 min |
| Melting curve analysis | 65 °C | 0:05 min |
|  | 95 °C | 0:05 min |

**RNA-seq analysis**

The libraries were sequenced on an Illumina HiSeqX Ten platform, and 150 bp paired-end reads were produced. Trimmomatic (7) was used to handle raw data in fastq format, and the low-quality reads were eliminated. For each sample, about 6.5 G clean reads were retained for further analyses. Bowtie2 was used to map the clean reads to the reference BSF genome database (8). The edgeR program was used to perform differential expression analysis at the threshold of biological coefficient of variation (square-root-dispersion) < 0.4 and P < 0.05. Annotations from the Kyoto Encyclopedia of Genes and Genomes (KEGG) were used to identify functions of the differentially expressed genes (9).

**References:**

1. Chen S, Hou C, Bi H, Wang Y, Xu J, Li M, James AA, Huang Y, Tan A. 2017. Transgenic Clustered Regularly Interspaced Short Palindromic Repeat/Cas9-Mediated Viral Gene Targeting for Antiviral Therapy of Bombyx mori Nucleopolyhedrovirus. J Virol 91.

2. Lenski RE, Bouma JE. 1987. Effects of Segregation and Selection on Instability of Plasmid Pacyc184 in Escherichia-Coli-B. Journal of Bacteriology 169:5314-5316.

3. Ainala SK, Seol E, Kim JR, Park S. 2017. Citrobacter amalonaticus Y19 for constitutive expression of carbon monoxide-dependent hydrogen-production machinery. Biotechnol Biofuels 10:80.

4. Gao Z, Deng W, Zhu F. 2019. Reference gene selection for quantitative gene expression analysis in black soldier fly (Hermetia illucens). PLoS One 14:e0221420.

5. Suzuki MT, Taylor LT, DeLong EF. 2000. Quantitative analysis of small-subunit rRNA genes in mixed microbial populations via 5'-nuclease assays. Appl Environ Microbiol 66:4605-14.

6. Livak KJ, Schmittgen TD. 2001. Analysis of relative gene expression data using real-time quantitative PCR and the 2(-Delta Delta C(T)) Method. Methods 25:402-8.

7. Bolger AM, Lohse M, Usadel B. 2014. Trimmomatic: a flexible trimmer for Illumina sequence data. Bioinformatics 30:2114-20.

8. Generalovic TN, McCarthy SA, Warren IA, Wood JMD, Torrance J, Sims Y, Quail M, Howe K, Pipan M, Durbin R, Jiggins CD. 2021. A high-quality, chromosome-level genome assembly of the Black Soldier Fly (Hermetia illucens L.). G3 (Bethesda) 11.

9. Kanehisa M, Araki M, Goto S, Hattori M, Hirakawa M, Itoh M, Katayama T, Kawashima S, Okuda S, Tokimatsu T, Yamanishi Y. 2008. KEGG for linking genomes to life and the environment. Nucleic Acids Res 36:D480-4.
