# Supplementary material for: A gut commensal bacterium promotes black soldier fly larval growth and development partly via modulation of intestinal protein metabolism
Source: mBio. 2023 Sep 14;14(5):e01174-23. doi: 10.1128/mbio.01174-23 (PMC10653789; doi:10.1128/mbio.01174-23)
Supplement: Text S2 — Sequence and annotation of plasmids generated in the study. [file mbio.01174-23-s0002.docx]

**Text S2** The sequence and annotation of plasmids generated in the study.

**The sequence of plasmid pDSRK-dsNS-hok (3862 bp)**

tcgcgcgtttcggtgatgacggtgactagtcttggactcctgttgatagatccagtaatgacctcagaactccatctggatttgttcagaacgctcggttgccgccgggcgttttttattggtgagaatccaggggtcccaacaaactccgggaggcagcgtgatgcggcaacaatcacacggatttcccgtgaacggtctgaatgagcggattattttcagggaaagtgagtgtggtcagcgtgcaggtatatgggctatgatgtgcccggcgcttgaggctttctgcctcatgacgtgaaggtggtttgttgccgtgttgtgtggcagaaagaagatagccccgtagtaagttaattttcattaaccaccacgaggcatccctatgtctagtccacatcaggatagcctcttaccgcgctttgcgcaaggagaagaaggccatgaaactaccacgaagttcccttgtctggtgtgtgttgatcgtgtgtctcacactgttgatattcacttatctgacacgaaaatcgctgtgcgagattcgttacagagacggacacagggaggtggcggctttcatggcttacgaatccggtaagtagcaacctggaggcgggcgcaggcccgccttttcaggactgatgctggtctgactactgaagcgcctttataaaggggctgctggttcgccggtagcccctttctccttgctgatgttgtatttaaatgggtgtcggggctggcttaactatgcggcatcagagcagattgtactgagagtgcaccatatgcctagggagagcgttcaccgacaaacaacagataaaacgaaaggcccagtctttcgactgagcctttcgttttatttgatgcctggctttggcagtttattcttgacatgtagtgagggggctggtataatcacatagtactgttgagctcTCATCGTGAATACTCCAGCCAAGTTCGCATAAAAGATTGTAACCAGCCACCGTGTGCGGTAGTGAAAGGAACGACGGCCAACTTTCAGGTAGACTTCGAGGCAGCCTTCCCAATAAAAATTATGACAACAAAGGTTCGAGCTACTGTCCTGGGACTCACCGTCAACTATCCACTTGACGACGAGCAGGCGAATACATGCGCCCATTTGCTGTATGGGAGCTATTGTCCGCTAGATAGAGGCGAGGATATTACATACAATTTTGACTTTCCGGTCGGAAACTCATATCCGGAAATTGGAGTCAATGTTGAAGTTAGCTTGGAAGATGAATCAAAAAAGGTGGTGACATGCTTTAATGTTGACAGTCTAGTCACGGATATCaacagtactatgtgattataccagccccctcactacatgtcaagaataaactgccaaaggtaatcgttaatccgcaaataacgtaaaaacccgcttcggcgggtttttttatggggggagtttagggaaagagcatttgtcacctagggctcttccgcttcctcgctcactgactcgctgcgctcggtcgttcggctgcggcgagcggtatcagctcactcaaaggcggtaatacggttatccacagaatcaggggataacgcaggaaagaacatgtgagcaaaaggccagcaaaaggccaggaaccgtaaaaaggccgcgttgctggcgtttttccataggctccgcccccctgacgagcatcacaaaaatcgacgctcaagtcagaggtggcgaaacccgacaggactataaagataccaggcgtttccccctggaagctccctcgtgcgctctcctgttccgaccctgccgcttaccggatacctgtccgcctttctcccttcgggaagcgtggcgctttctcatagctcacgctgtaggtatctcagttcggtgtaggtcgttcgctccaagctgggctgtgtgcacgaaccccccgttcagcccgaccgctgcgccttatccggtaactatcgtcttgagtccaacccggtaagacacgacttatcgccactggcagcagccactggtaacaggattagcagagcgaggtatgtaggcggtgctacagagttcttgaagtggtggcctaactacggctacactagaagaacagtatttggtatctgcgctctgctgaagccagttaccttcggaaaaagagttggtagctcttgatccggcaaacaaaccaccgctggtagcggtggtttttttgtttgcaagcagcagattacgcgcagaaaaaaaggatctcaagaagatcctttgatcttttctacggggtctgacgctcagtggaacgaaaactcacgttaagggattttggtcatgagattatcaaaaaggatcttcacctagatccttttaaattaaaaatgaagttttaaatcaatctaaagtatatatgagtaaacttggtctgacagttaccaatgcttaatcagtgaggcacctatctcagcgatctgtctatttcgttcatccatagttgcctgactccccgtcgtgtagataactacgatacgggagggcttaccatctggccccagtgctgcaatgataccgcgagacccacgctcaccggctccagatttatcagcaataaaccagccagccggaagggccgagcgcagaagtggtcctgcaactttatccgcctccatccagtcttagactgggcggttttatggacagcaagcgaaccggaattgccagctggggcgccctctggtaaggttgggaagccctgcaaagtaaactggatggctttcttgccgccaaggatctgatggcgcaggggatcaagatctgatcaagagacaggatgaggatcgtttcgcatgattgaacaagatggattgcacgcaggttctccggccgcttgggtggagaggctattcggctatgactgggcacaacagacaatcggctgctctgatgccgccgtgttccggctgtcagcgcaggggcgcccggttctttttgtcaagaccgacctgtccggtgccctgaatgaactgcaggacgaggcagcgcggctatcgtggctggccacgacgggcgttccttgcgcagctgtgctcgacgttgtcactgaagcgggaagggactggctgctattgggcgaagtgccggggcaggatctcctgtcatctcaccttgctcctgccgagaaagtatccatcatggctgatgcaatgcggcggctgcatacgcttgatccggctacctgcccattcgaccaccaagcgaaacatcgcatcgagcgagcacgtactcggatggaagccggtcttgtcgatcaggatgatctggacgaagagcatcaggggctcgcgccagccgaactgttcgccaggctcaaggcgcgcatgcccgacggcgaggatctcgtcgtgacccatggcgatgcctgcttgccgaatatcatggtggaaaatggccgcttttctggattcatcgactgtggccggctgggtgtggcggaccgctatcaggacatagcgttggctacccgtgatattgctgaagagcttggcggcgaatgggctgaccgcttcctcgtgctttacggtatcgccgctcccgattcgcagcgcatcgccttctatcgccttcttgacgagttcttctgagcgggactctggggttcgaaatgaccgaccaagcgacgcccaacctgccatcacgagatttattgaagcatttatcagggttattgtctcatgagcggatacatatttgaatgtatttagaaaaataaacaaataggggttccgcgcacatttccccgaaaagtgccacctgacgtctaagaaaccattattatcatgacattaacctataaaaataggcgtatcacgaggccctttcgtc

Plasmid annotation: nucleotide sequence 176-703 is the hok/sok system; nucleotide sequence 798-877 is the rrnB T1 terminator; nucleotide sequence 878-936 and 1322-1380 are the sequences of the two copies of the convergent cp25 promoter; nucleotide sequence 937-1321 is the synthetized non-sense sequence which can be replaced with dsRNA targeting sequence; nucleotide sequence 1381-1463 is the rpoC terminator; nucleotide sequence 1639-2253 is the replicon of pUC57; nucleotide sequence 2827-3621 is the kanamycin resistance gene.

**The sequence of plasmid pDSRK-dsGFP-hok (3848 bp)**

tcgcgcgtttcggtgatgacggtgactagtcttggactcctgttgatagatccagtaatgacctcagaactccatctggatttgttcagaacgctcggttgccgccgggcgttttttattggtgagaatccaggggtcccaacaaactccgggaggcagcgtgatgcggcaacaatcacacggatttcccgtgaacggtctgaatgagcggattattttcagggaaagtgagtgtggtcagcgtgcaggtatatgggctatgatgtgcccggcgcttgaggctttctgcctcatgacgtgaaggtggtttgttgccgtgttgtgtggcagaaagaagatagccccgtagtaagttaattttcattaaccaccacgaggcatccctatgtctagtccacatcaggatagcctcttaccgcgctttgcgcaaggagaagaaggccatgaaactaccacgaagttcccttgtctggtgtgtgttgatcgtgtgtctcacactgttgatattcacttatctgacacgaaaatcgctgtgcgagattcgttacagagacggacacagggaggtggcggctttcatggcttacgaatccggtaagtagcaacctggaggcgggcgcaggcccgccttttcaggactgatgctggtctgactactgaagcgcctttataaaggggctgctggttcgccggtagcccctttctccttgctgatgttgtatttaaatgggtgtcggggctggcttaactatgcggcatcagagcagattgtactgagagtgcaccatatgcctagggagagcgttcaccgacaaacaacagataaaacgaaaggcccagtctttcgactgagcctttcgttttatttgatgcctggctttggcagtttattcttgacatgtagtgagggggctggtataatcacatagtactgttccacaagttcagcgtgtccggcgagggcgagggcgatgccacctacggcaagctgaccctgaagttcatctgcaccaccggcaagctgcccgtgccctggcccaccctcgtgaccaccctgacctacggcgtgcagtgcttcagccgctaccccgaccacatgaagcagcacgacttcttcaagtccgccatgcccgaaggctacgtccaggagcgcaccatcttcttcaaggacgacggcaactacaagacccgcgccgaggtgaagttcgagggcgacaccctggtgaaccgcatcgagctgaagggcatcgacttcaaggaggacggcaacatcctggggcacaagctggagtacaactacaacagccaacagtactatgtgattataccagccccctcactacatgtcaagaataaactgccaaaggtaatcgttaatccgcaaataacgtaaaaacccgcttcggcgggtttttttatggggggagtttagggaaagagcatttgtcacctagggctcttccgcttcctcgctcactgactcgctgcgctcggtcgttcggctgcggcgagcggtatcagctcactcaaaggcggtaatacggttatccacagaatcaggggataacgcaggaaagaacatgtgagcaaaaggccagcaaaaggccaggaaccgtaaaaaggccgcgttgctggcgtttttccataggctccgcccccctgacgagcatcacaaaaatcgacgctcaagtcagaggtggcgaaacccgacaggactataaagataccaggcgtttccccctggaagctccctcgtgcgctctcctgttccgaccctgccgcttaccggatacctgtccgcctttctcccttcgggaagcgtggcgctttctcatagctcacgctgtaggtatctcagttcggtgtaggtcgttcgctccaagctgggctgtgtgcacgaaccccccgttcagcccgaccgctgcgccttatccggtaactatcgtcttgagtccaacccggtaagacacgacttatcgccactggcagcagccactggtaacaggattagcagagcgaggtatgtaggcggtgctacagagttcttgaagtggtggcctaactacggctacactagaagaacagtatttggtatctgcgctctgctgaagccagttaccttcggaaaaagagttggtagctcttgatccggcaaacaaaccaccgctggtagcggtggtttttttgtttgcaagcagcagattacgcgcagaaaaaaaggatctcaagaagatcctttgatcttttctacggggtctgacgctcagtggaacgaaaactcacgttaagggattttggtcatgagattatcaaaaaggatcttcacctagatccttttaaattaaaaatgaagttttaaatcaatctaaagtatatatgagtaaacttggtctgacagttaccaatgcttaatcagtgaggcacctatctcagcgatctgtctatttcgttcatccatagttgcctgactccccgtcgtgtagataactacgatacgggagggcttaccatctggccccagtgctgcaatgataccgcgagacccacgctcaccggctccagatttatcagcaataaaccagccagccggaagggccgagcgcagaagtggtcctgcaactttatccgcctccatccagtcttagactgggcggttttatggacagcaagcgaaccggaattgccagctggggcgccctctggtaaggttgggaagccctgcaaagtaaactggatggctttcttgccgccaaggatctgatggcgcaggggatcaagatctgatcaagagacaggatgaggatcgtttcgcatgattgaacaagatggattgcacgcaggttctccggccgcttgggtggagaggctattcggctatgactgggcacaacagacaatcggctgctctgatgccgccgtgttccggctgtcagcgcaggggcgcccggttctttttgtcaagaccgacctgtccggtgccctgaatgaactgcaggacgaggcagcgcggctatcgtggctggccacgacgggcgttccttgcgcagctgtgctcgacgttgtcactgaagcgggaagggactggctgctattgggcgaagtgccggggcaggatctcctgtcatctcaccttgctcctgccgagaaagtatccatcatggctgatgcaatgcggcggctgcatacgcttgatccggctacctgcccattcgaccaccaagcgaaacatcgcatcgagcgagcacgtactcggatggaagccggtcttgtcgatcaggatgatctggacgaagagcatcaggggctcgcgccagccgaactgttcgccaggctcaaggcgcgcatgcccgacggcgaggatctcgtcgtgacccatggcgatgcctgcttgccgaatatcatggtggaaaatggccgcttttctggattcatcgactgtggccggctgggtgtggcggaccgctatcaggacatagcgttggctacccgtgatattgctgaagagcttggcggcgaatgggctgaccgcttcctcgtgctttacggtatcgccgctcccgattcgcagcgcatcgccttctatcgccttcttgacgagttcttctgagcgggactctggggttcgaaatgaccgaccaagcgacgcccaacctgccatcacgagatttattgaagcatttatcagggttattgtctcatgagcggatacatatttgaatgtatttagaaaaataaacaaataggggttccgcgcacatttccccgaaaagtgccacctgacgtctaagaaaccattattatcatgacattaacctataaaaataggcgtatcacgaggccctttcgtc

Plasmid annotation: nucleotide sequence 176-703 is the hok/sok system; nucleotide sequence 798-877 is the rrnB T1 terminator; nucleotide sequence 878-936 and 1308-1366 are the sequences of the two copies of the convergent cp25 promoter; nucleotide sequence 937-1307 is the dsRNA targeting region of EGFP; nucleotide sequence 1367-1449 is the rpoC terminator; nucleotide sequence 1625-2239 is the replicon of pUC57; nucleotide sequence 2813-3607 is the kanamycin resistance gene.

**The sequence of plasmid pBBR1MCS-2-cp25EGFP (5552 bp)**

ctcgggccgtctcttgggcttgatcggccttcttgcgcatctcacgcgctcctgcggcggcctgtagggcaggctcatacccctgccgaaccgcttttgtcagccggtcggccacggcttccggcgtctcaacgcgctttgagattcccagcttttcggccaatccctgcggtgcataggcgcgtggctcgaccgcttgcgggctgatggtgacgtggcccactggtggccgctccagggcctcgtagaacgcctgaatgcgcgtgtgacgtgccttgctgccctcgatgccccgttgcagccctagatcggccacagcggccgcaaacgtggtctggtcgcgggtcatctgcgctttgttgccgatgaactccttggccgacagcctgccgtcctgcgtcagcggcaccacgaacgcggtcatgtgcgggctggtttcgtcacggtggatgctggccgtcacgatgcgatccgccccgtacttgtccgccagccacttgtgcgccttctcgaagaacgccgcctgctgttcttggctggccgacttccaccattccgggctggccgtcatgacgtactcgaccgccaacacagcgtccttgcgccgcttctctggcagcaactcgcgcagtcggcccatcgcttcatcggtgctgctggccgcccagtgctcgttctctggcgtcctgctggcgtcagcgttgggcgtctcgcgctcgcggtaggcgtgcttgagactggccgccacgttgcccattttcgccagcttcttgcatcgcatgatcgcgtatgccgccatgcctgcccctcccttttggtgtccaaccggctcgacgggggcagcgcaaggcggtgcctccggcgggccactcaatgcttgagtatactcactagactttgcttcgcaaagtcgtgaccgcctacggcggctgcggcgccctacgggcttgctctccgggcttcgccctgcgcggtcgctgcgctcccttgccagcccgtggatatgtggacgatggccgcgagcggccaccggctggctcgcttcgctcggcccgtggacaaccctgctggacaagctgatggacaggctgcgcctgcccacgagcttgaccacagggattgcccaccggctacccagccttcgaccacatacccaccggctccaactgcgcggcctgcggccttgccccatcaatttttttaattttctctggggaaaagcctccggcctgcggcctgcgcgcttcgcttgccggttggacaccaagtggaaggcgggtcaaggctcgcgcagcgaccgcgcagcggcttggccttgacgcgcctggaacgacccaagcctatgcgagtgggggcagtcgaagggcgaagcccgcccgcctgccccccgagcctcacggcggcgagtgcgggggttccaagggggcagcgccaccttgggcaaggccgaaggccgcgcagtcgatcaacaagccccggaggggccactttttgccggagggggagccgcgccgaaggcgtgggggaaccccgcaggggtgcccttctttgggcaccaaagaactagatatagggcgaaatgcgaaagacttaaaaatcaacaacttaaaaaaggggggtacgcaacagctcattgcggcaccccccgcaatagctcattgcgtaggttaaagaaaatctgtaattgactgccacttttacgcaacgcataattgttgtcgcgctgccgaaaagttgcagctgattgcgcatggtgccgcaaccgtgcggcacccctaccgcatggagataagcatggccacgcagtccagagaaatcggcattcaagccaagaacaagcccggtcactgggtgcaaacggaacgcaaagcgcatgaggcgtgggccgggcttattgcgaggaaacccacggcggcaatgctgctgcatcacctcgtggcgcagatgggccaccagaacgccgtggtggtcagccagaagacactttccaagctcatcggacgttctttgcggacggtccaatacgcagtcaaggacttggtggccgagcgctggatctccgtcgtgaagctcaacggccccggcaccgtgtcggcctacgtggtcaatgaccgcgtggcgtggggccagccccgcgaccagttgcgcctgtcggtgttcagtgccgccgtggtggttgatcacgacgaccaggacgaatcgctgttggggcatggcgacctgcgccgcatcccgaccctgtatccgggcgagcagcaactaccgaccggccccggcgaggagccgcccagccagcccggcattccgggcatggaaccagacctgccagccttgaccgaaacggaggaatgggaacggcgcgggcagcagcgcctgccgatgcccgatgagccgtgttttctggacgatggcgagccgttggagccgccgacacgggtcacgctgccgcgccggtagcacttgggttgcgcagcaacccgtaagtgcgctgttccagactatcggctgtagccgcctcgccgccctataccttgtctgcctccccgcgttgcgtcgcggtgcatggagccgggccacctcgacctgaatggaagccggcggcacctcgctaacggattcaccgtttttatcaggctctgggaggcagaataaatgatcatatcgtcaattattacctccacggggagagcctgagcaaactggcctcaggcatttgagaagcacacggtcacactgcttccggtagtcaataaaccggtaaaccagcaatagacataagcggctatttaacgaccctgccctgaaccgacgaccgggtcgaatttgctttcgaatttctgccattcatccgcttattatcacttattcaggcgtagcaaccaggcgtttaagggcaccaataactgccttaaaaaaattacgccccgccctgccactcatcgcagtacggcctattggttaaaaaatgagctgatttaacaaaaatttaacgcgaattttaacaaaatattaacgcttacaatttccattcgccattcaggctgcgcaactgttgggaagggctttggcagtttattcttgacatgtagtgagggggctggtataatcacatagtactgttgattcattaaagaggagaaaggtaccatggtgagcaagggcgaggagctgttcaccggggtggtgcccatcctggtcgagctggacggcgacgtaaacggccacaagttcagcgtgtccggcgagggcgagggcgatgccacctacggcaagctgaccctgaagttcatctgcaccaccggcaagctgcccgtgccctggcccaccctcgtgaccaccctgacctacggcgtgcagtgcttcagccgctaccccgaccacatgaagcagcacgacttcttcaagtccgccatgcccgaaggctacgtccaggagcgcaccatcttcttcaaggacgacggcaactacaagacccgcgccgaggtgaagttcgagggcgacaccctggtgaaccgcatcgagctgaagggcatcgacttcaaggaggacggcaacatcctggggcacaagctggagtacaactacaacagccacaacgtctatatcatggccgacaagcagaagaacggcatcaaggtgaacttcaagatccgccacaacatcgaggacggcagcgtgcagctcgccgaccactaccagcagaacacccccatcggcgacggccccgtgctgctgcccgacaaccactacctgagcacccagtccgccctgagcaaagaccccaacgagaagcgcgatcacatggtcctgctggagttcgtgaccgccgccgggatcactctcggcatggacgagctgtacaagtaaaacagtactatgtgattataccagccccctcactacatgtcaagaataaactgccaaaggtaatcgttaatccgcaaataacgtaaaaacccgcttcggcgggtttttttatggggggagtttagggaaagagcatttgtcaaaactgttgtaattcattaagcattctgccgacatggaagccatcacaaacggcatgatgaacctgaatcgccagcggcatcagcaccttgtcgccttgcgtataatatttgcccatgggggtgggcgaagaactccagcatgagatccccgcgctggaggatcatccagccggcgtcccggaaaacgattccgaagcccaacctttcatagaaggcggcggtggaatcgaaatctcgtgatggcaggttgggcgtcgcttggtcggtcatttcgaaccccagagtcccgctcagaagaactcgtcaagaaggcgatagaaggcgatgcgctgcgaatcgggagcggcgataccgtaaagcacgaggaagcggtcagcccattcgccgccaagctcttcagcaatatcacgggtagccaacgctatgtcctgatagcggtccgccacacccagccggccacagtcgatgaatccagaaaagcggccattttccaccatgatattcggcaagcaggcatcgccatgggtcacgacgagatcctcgccgtcgggcatgcgcgccttgagcctggcgaacagttcggctggcgcgagcccctgatgctcttcgtccagatcatcctgatcgacaagaccggcttccatccgagtacgtgctcgctcgatgcgatgtttcgcttggtggtcgaatgggcaggtagccggatcaagcgtatgcagccgccgcattgcatcagccatgatggatactttctcggcaggagcaaggtgagatgacaggagatcctgccccggcacttcgcccaatagcagccagtcccttcccgcttcagtgacaacgtcgagcacagctgcgcaaggaacgcccgtcgtggccagccacgatagccgcgctgcctcgtcctgcagttcattcagggcaccggacaggtcggtcttgacaaaaagaaccgggcgcccctgcgctgacagccggaacacggcggcatcagagcagccgattgtctgttgtgcccagtcatagccgaatagcctctccacccaagcggccggagaacctgcgtgcaatccatcttgttcaatcatgcgaaacgatcctcatcctgtctcttgatcagatcttgatcccctgcgccatcagatccttggcggcaagaaagccatccagtttactttgcagggcttcccaaccttaccagagggcgccccagctggcaattccggttcgcttgctgtccataaaaccgcccagtctagctatcgccatgtaagcccactgcaagctacctgctttctctttgcgcttgcgttttcccttgtccagatagcccagtagctgacattcatcccaggtggcacttttcggggaaatgtgcgcgcccgcgttcctgctggcgctgggcctgtttctggcgctggacttcccgctgttccgtcagcagcttttcgcccacggccttgatgatcgcggcggccttggcctgcatatcccgattcaacggccccagggcgtccagaacgggcttcaggcgctcccgaaggt

Plasmid annotation: nucleotide sequence 1023-2457 includes pBBR1 oriV (replication origin) and pBBR1 Rep (replication protein); nucleotide sequence 3063-3121 is the cp25 promoter driving the expression EGFP; nucleotide sequence 3130-3141 is the ribosome binding site; nucleotide sequence 3148-3867 is the coding sequence of EGFP; nucleotide sequence 4301-5095 is the kanamycin resistance gene.

**The sequence of plasmid pKOVK (9129 bp)**

ccctttcgtcttcgaataaatacctgtgacggaagatcacttcgcagaataaataaatcctggtgtccctgttgataccgggaagccctgggccaacttttggctagactgggcggttttatggacagcaagcgaaccggaattgccagctggggcgccctctggtaaggttgggaagccctgcaaagtaaactggatggctttcttgccgccaaggatctgatggcgcaggggatcaagatctgatcaagagacaggatgaggatcgtttcgcatgattgaacaagatggattgcacgcaggttctccggccgcttgggtggagaggctattcggctatgactgggcacaacagacaatcggctgctctgatgccgccgtgttccggctgtcagcgcaggggcgcccggttctttttgtcaagaccgacctgtccggtgccctgaatgaactgcaggacgaggcagcgcggctatcgtggctggccacgacgggcgttccttgcgcagctgtgctcgacgttgtcactgaagcgggaagggactggctgctattgggcgaagtgccggggcaggatctcctgtcatctcaccttgctcctgccgagaaagtatccatcatggctgatgcaatgcggcggctgcatacgcttgatccggctacctgcccattcgaccaccaagcgaaacatcgcatcgagcgagcacgtactcggatggaagccggtcttgtcgatcaggatgatctggacgaagagcatcaggggctcgcgccagccgaactgttcgccaggctcaaggcgcgcatgcccgacggcgaggatctcgtcgtgacccatggcgatgcctgcttgccgaatatcatggtggaaaatggccgcttttctggattcatcgactgtggccggctgggtgtggcggaccgctatcaggacatagcgttggctacccgtgatattgctgaagagcttggcggcgaatgggctgaccgcttcctcgtgctttacggtatcgccgctcccgattcgcagcgcatcgccttctatcgccttcttgacgagttcttctgagcgggactctggggttcgaaatgaccgaccaagcgacgcccaacctgccatcacgagattttccctgggtgagtttcaccagttttgatttaaacgtggccaatatggacaacttcttcgcccccgttttcaccatgggcaaatattatacgcaaggcgacaaggtgctgatgccgctggcgattcaggttcatcatgccgtttgtgatggcttccatgtcggcagaatgcttaatgaattacaacagtactgcgatgagtggcagggcggggcgtaatttttttaaggcagttattggtgcccttaaacgcctggttgctacgcctgaataagtgataataagcggatgaatggcagaaattcgaaagcaaattcgacccggtcgtcggttcagggcagggtcgttaaatagccgcttatgtctattgctggtttaatcggtacccggggatcgcggccgccccgaacccaactgatgcagtatcccgggcgctacaacatgatctcagtgatgttatcggtcaggaacaaggaaagcgaggactggaaattaccgctgctggcgggcacaaccttttactgattgggccgccgggaacaggtaaaacaatgctcgccagccgtattaatggcctgttgccagatttaagcaatgaagaggcactggagagtgctgcgatattaagtctggtaaatgctgaatcagtacaaaaacaatggcggcagcgcccgttccgctcacctcatcacagtgcatcgttaactgcgatggtaggcggtggcgcaattccagggcccggtgaaatttcgctggcgcataacggcgtgctttttcttgatgagctacctgaatttgaacggcgtacaccggatgccttgcgagagccgattgaatccgggcagatccatctttcacgcacacgagcaaaaataacctatccagcccgtttccagcttgttgcagcgatgaatcccagccctaccgggcattatcagggaaaccataaccgctgcacgccagaacagacattacgttatctcaaccggctctcggggccctttctcgaccgcttcgatctctcactggagatcccattaccaccccccggcactttgagtaaaacggtagtgccgggagaaagcagcgccaccattaaacaacgcgtaatggccgccagagagcgccaatttaagcggcagaataaactgaatgcctggctggatagtccggaaatacgccaattctgcaaacttgagagcgaagatgcgatgtggttggaaggaacactgatccatctggggttatcgattcgtgcctggcagcggttattgaaagttgcacgaaccattgctgatattgatcagtctgacattatcacacgtcagcatttgcaggaggcagttagctatcgagcgattgaccgtttgctcatccatctgcagaaactactgacataaaaaaagggcatttcgccctttttattaatcgtcagaatcggtgtagtcttcagcaccttcaacctgcggtttaccgcggaaagggtgtgaaaacgttttggacgcttaatacgcgtcatatacttggaccacacgcgttctgcttctgtcactggctcacgttcgccacggcatactgctacgaagagtttctcttcctcggtaaccggctcgcgtttgccaagatccaactcattgaaggcataaccatgacgctcaagcagttgtgcctctttgatggtgaaatcaccatgacgagagaatccacgtggataatgtttattgtcgaaatatcgattagtcgtcgtaaagctttccgccatcctgcacgctcctaattctttgaccgagctagttatggcgcggagtattagttacgcttgacagagtgtaaaacaaaacatttaaatcataacgacaaataattttgcggagagcactgtggatgttataaatttggagtgtgaaggttattgcgtgtggtaaaacagctgcatgaacgccagcttgatcttcttattaccactgaagcgcccaaaatggacgaatttagtagtcagttgctgggatatttcactttagcgctttataccagtgccccttcaaaactaaagggagatcttaattatctgcgacttgagtgggggccagattttcaacagcatgaggcaggtttgatcggtgctgacgaagtgcccattctgacaaccagttctgctgaactggcacagcaacagattgcgatgcttaatggttgcacctggctacccgtcagctgggcgcgtaaaaaaggcggcctgcataccgttgtcgatagcacaacactttcacggccgctttatgccatatggctgcaaaatagcgataaaaatgcgttgattcgcgatcttttgaaaattaacgtgctggatgaagtgtattaatatgaatggctggcaaggatgccggtagaaggatttacttcggagagggttatttcagataaaaaaaatccttacgtttcgctaaggatgatttctggcaggggcggagagactcgaactcccaacacccggttttggagaccggtgctctaccaattgaactacgcccctaattagggtggcggaacggacgggactcgaacccgcgaccccctgcgtgacaggcaggtattctaaccgactgaactaccgctccaccgaattcttttacaaccaccggttttatgaccggcttactgcttaatttgatgcctggcagttccctactctcgcatggggagaccccacactaccatcggcgctacggcgtttcacttctgagttcggcatggggtcaggtgggaccaccgcgctacggccgccaggcaaattctgtttcatcagaccgcttctgcgttctgatttaatctgtatcaggctgaaaatcttctctcatccgccaaaacatcttcggcgttgtaaggttaagcctcacggttcattagtaccggttagctcaacgcatcgctgcgcttacacacccggcctatcaacgtcgtcgtcttcaacgttccttcaggactctcaaggagtcagggagaactcatctcggggcaagtttcgtgcttagatgctttcagcacttatctcttccgcatttagctaccgggcagtgccattggcatgacaacccgaacaccagtgatgcgtccactccggtcctctcgtactaggagcagcccccctcagttctccagcgcccacggcagatagggaccgaactgtctcacgacgttctaaacccagctcgcgtaccactttaaatggcgaacagccatacccttgggacctacttcagctccaggatgtgatgagccgacatcgaggtgccaaacaccgccgtcgatatgaactcttgggcggtatcagcctgttatccccggagtaccttttatccgttgagcgatggcccttccattcagaaccaccggatcactatgacctgctttcgcacctgggatcctctagagtcgaccggagaatggcgcgcgccctgtagcggcgcattaagcgcggcgggtgtggtggttacgcgcagcgtgaccgctacacttgccagcgccctagcgcccgctcctttcgctttcttcccttcctttctcgccacgttcgccggctttccccgtcaagctctaaatcgggggctccctttagggttccgatttagtgctttacggcacctcgaccccaaaaaacttgattagggtgatggttcacgtagtgggccatcgccctgatagacggtttttcgccctttgacgttggagtccacgttctttaatagtggactcttgttccaaactggaacaacactcaaccctatctcggtctattcttttgatttataagggattttgccgatttcggcctattggttaaaaaatgagctgatttaacaaaaatttaacgcgaattttaacaaaatattaacgcttacaatttaggtggcacttttcggggaaatgtgcgcggaacccctatttgtttatttttctaaatacattcaaatatgtatccgctcatnncangatcctttttaacccatcacatatacctgccgttcactattatttagtgaaatgagatattatgatattttctgaattgtgattaaaaaggcaactttatgcccatgcaacagaaactataaaaaatacagagaatgaaaagaaacagatagattttttagttctttaggcccgtagtctgcaaatccttttatgattttctatcaaacaaaagaggaaaatagaccagttgcaatccaaacgagagtctaatagaatgaggtcgaaaagtaaatcgcgcgggtttgttactgataaagcaggcaagacctaaaatgtgtaaagggcaaagtgtatactttggcgtcaccccttacatattttaggtctttttttattgtgcgtaactaacttgccatcttcaaacaggagggctggaagaagcagaccgctaacacagtacataaaaaaggagacatgaacgatgaacatcaaaaagtttgcaaaacaagcaacagtattaacctttactaccgcactgctggcaggaggcgcaactcaagcgtttgcgaaagaaacgaaccaaaagccatataaggaaacatacggcatttcccatattacacgccatgatatgctgcaaatccctgaacagcaaaaaaatgaaaaatatcaagttcctgagttcgattcgtccacaattaaaaatatctcttctgcaaaaggcctggacgtttgggacagctggccattacaaaacgctgacggcactgtcgcaaactatcacggctaccacatcgtctttgcattagccggagatcctaaaaatgcggatgacacatcgatttacatgttctatcaaaaagtcggcgaaacttctattgacagctggaaaaacgctggccgcgtctttaaagacagcgacaaattcgatgcaaatgattctatcctaaaagaccaaacacaagaatggtcaggttcagccacagttacatctgacggaaaaatccgtttattctacactgatttctccggtaaacattacggcaaacaaacactgacaactgcacaagttaacgtatcagcatcagacagctctttgaacatcaacggtgtagaggattataaatcaatctttgacggtgacggaaaaacgtatcaaaatgtacagcagttcatcgatgaaggcaactacagctcaggcgacaaccatacgctgagagatcctcactacgtagaagataaaggccacaaatacttagtatttgaagcaaacactggaactgaagatggctaccaaggcgaagaatctttatttaacaaagcatactatggcaaaagcacatcattcttccgtcaagaaagtcaaaaacttctgcaaagcgataaaaaacgcacggctgagttagcaaacggcgctctcggtatgattgagctaaacgatgattacacactgaaaaaagtgatgaaaccgctgattgcatctaacacagtaacagatgaaattgaacgcgcgaacgtctttaaaatgaacggcaaatggtacctgttcactgactcccgcggatcaaaaatgacgattgacggcattacgtctaacgatatttacatgcttggttatgtttctaattctttaactggcccatacaagccgctgaacaaaactggccttgtgttaaaaatggatcttgatcctaacgatgtaacctttacttactcacacttcgctgtacctcaagcgaaaggaaacaatgtcgtgattacaagctatatgacaaacagaggattctacgcagacaaacaatcaacgtttgcgccaagcttcctgctgaacatcaaaggcaagaaaacatctgttgtcaaagacagcatccttgagcaaggacaattaacagttaacaaataaaaacgcaaaagaaaatgccgatattgactaccggaagcagtgtgaccgtgtgcttctcaaatgcctgattcaggctgtctatgtgtgactgttgagctgtaacaagttgtctcaggtgttcaatttcatgttctagttgctttgttttactggtttcacctgttctgttaggtgttacatgctgttcatctgttacattgtcgatctgttcatggtgaacagctttaaatgcaccaaaaactcgtaaaagctctgatgtatctatcttttttacaccgttttcatctgtgcatatggacagttttccctttgatatctaacggtgaacagttgttctacttttgtttgttagtcttgatgcttcactgatagatacaagagccataagaacctcagatccttccgtatttagccagtatgttctctagtgtggttcgttgtttttgcgtgagccatgagaacgaaccattgagatcatgcttactttgcatgtcactcaaaaattttgcctcaaaactggtgagctgaatttttgcagttaaagcatcgtgtagtgtttttcttagtccgttacgtaggtaggaatctgatgtaatggttgttggtattttgtcaccattcatttttatctggttgttctcaagttcggttacgagatccatttgtctatctagttcaacttggaaaatcaacgtatcagtcgggcggcctcgcttatcaaccaccaatttcatattgctgtaagtgtttaaatctttacttattggtttcaaaacccattggttaagccttttaaactcatggtagttattttcaagcattaacatgaacttaaattcatcaaggctaatctctatatttgccttgtgagttttcttttgtgttagttcttttaataaccactcataaatcctcatagagtatttgttttcaaaagacttaacatgttccagattatattttatgaatttttttaactggaaaagataaggcaatatctcttcactaaaaactaattctaatttttcgcttgagaacttggcatagtttgtccactggaaaatctcaaagcctttaaccaaaggattcctgatttccacagttctcgtcatcagctctctggttgctttagctaatacaccataagcattttccctactgatgttcatcatctgaacgtattggttataagtgaacgataccgtccgttctttcctcgtagggttttcaatcgtggggttgagtagtgccacacagcataaaattagcttggtttcatgctccgttaagtcatagcgactaatcgctagttcatttgctttgaaaacaactaattcagacatacatctcaattggtctaggtgattttaatcactataccaattgagatgggctagtcaatgataattactagtccttttcctttgagttgtgggtatctgtaaattctgctagacctttgctggaaaacttgtaaattctgctagaccctctgtaaattccgctagacctttgtgtgttttttttgtttatattcaagtggttataatttatagaataaagaaagaataaaaaaagataaaaagaatagatcccagccctgtgtataactcactactttagtcagttccgcagtattacaaaaggatgtcgtaaacgctgtttgctcctctacaaaacagaccttaaaaccctaaaggcttaagtagcaccctcgcaagctcgggcaaatcgctgaatattccttctgtctccgaccatccggcacctgagtcgctgtctttttcgtgacattcagtccgctgcgctcacggctctggcagtgaatgggggtaaatggcactacaggcgccttttatggattcatgcaaggaaactacccataatacaagaaaagcccgtcacgggcttctcagggcgttttatggcgggtctgctatgtggtgctatctgactttttgctgttcagcagttcctgccctctgattttccagtctgaccacttcggattatcccgtgacaggtcattcagactggctaatgcacccagtaaggcagcggtatcatcaacaggcttacccgtcttactgtcnggatcgacgctctcccttatgcgactcctgcat

Plasmid annotation: nucleotide sequence 275-1069 is the kanamycin resistance gene; nucleotide sequence 4578-5032 is the f1 bacteriophage origin of replication; nucleotide sequence 5136-7003 is the *sacB* gene expression cassette; nucleotide sequence 7396-8346 is the temperature-sensitive version of the RepA protein needed for replication with the pSC101 origin; nucleotide sequence 8394-8616 is the pSC101 origin.

**The sequence of plasmid pKOVK-Δ*rnc* (7109 bp)**

ccctttcgtcttcgaataaatacctgtgacggaagatcacttcgcagaataaataaatcctggtgtccctgttgataccgggaagccctgggccaacttttggctagactgggcggttttatggacagcaagcgaaccggaattgccagctggggcgccctctggtaaggttgggaagccctgcaaagtaaactggatggctttcttgccgccaaggatctgatggcgcaggggatcaagatctgatcaagagacaggatgaggatcgtttcgcatgattgaacaagatggattgcacgcaggttctccggccgcttgggtggagaggctattcggctatgactgggcacaacagacaatcggctgctctgatgccgccgtgttccggctgtcagcgcaggggcgcccggttctttttgtcaagaccgacctgtccggtgccctgaatgaactgcaggacgaggcagcgcggctatcgtggctggccacgacgggcgttccttgcgcagctgtgctcgacgttgtcactgaagcgggaagggactggctgctattgggcgaagtgccggggcaggatctcctgtcatctcaccttgctcctgccgagaaagtatccatcatggctgatgcaatgcggcggctgcatacgcttgatccggctacctgcccattcgaccaccaagcgaaacatcgcatcgagcgagcacgtactcggatggaagccggtcttgtcgatcaggatgatctggacgaagagcatcaggggctcgcgccagccgaactgttcgccaggctcaaggcgcgcatgcccgacggcgaggatctcgtcgtgacccatggcgatgcctgcttgccgaatatcatggtggaaaatggccgcttttctggattcatcgactgtggccggctgggtgtggcggaccgctatcaggacatagcgttggctacccgtgatattgctgaagagcttggcggcgaatgggctgaccgcttcctcgtgctttacggtatcgccgctcccgattcgcagcgcatcgccttctatcgccttcttgacgagttcttctgagcgggactctggggttcgaaatgaccgaccaagcgacgcccaacctgccatcacgagattttccctgggtgagtttcaccagttttgatttaaacgtggccaatatggacaacttcttcgcccccgttttcaccatgggcaaatattatacgcaaggcgacaaggtgctgatgccgctggcgattcaggttcatcatgccgtttgtgatggcttccatgtcggcagaatgcttaatgaattacaacagtactgcgatgagtggcagggcggggcgtaatttttttaaggcagttattggtgcccttaaacgcctggttgctacgcctgaataagtgataataagcggatgaatggcagaaattcgaaagcaaattcgacccggtcgtcggttcagggcagggtcgttaaatagccgcttatgtctattgctggtttaatcggtacccggggatcAGCCGCTGGCGACCTGGATTGTGCCGCCGGGCCAGTATTTCATGATGGGCGACAACCGCGATAACAGCGCGGACAGCCGTTACTGGGGCTTTGTCCCGGAAGCGAATCTGGTCGGTAAAGCCACTGCCATCTGGATGAGTTTCGACAAACAAGAAGGCGAATGGCCGACTGGCGTTCGTTTAAGCCGCATTGGTGGTATCCACTAATTGCCGCGAATCGTTCACGTTGTCGTCGTTATGACGGCAACGTGAATTATTTCTTCGATAAATCTGCCCAGGCTAACGACATCCCTCGTCGTTGTGTATAGAATATTCCCCCGAAGTTTCAGGTTGGCGCCTCCAGGCCGCCACGGCACACGAAACAGCGTTGGTCCCCCTATATGAGCGTACATAAGCTGCTTAACGCGGCCGGAAGGATGCTACATATATCAGGTCTGTTTCGTGTGCTGAATTGTTGACGCATTTATTTATTGGTATCGCATGAACCCCATCGTAATTAATAAACTGGAGCTGGAATGAGCGACGAAAAAACCTATTGCGGATTTATTGCCATCGTCGGTCGTCCGAATGTTGGCAAATCCACGCTGTTGAACAACCTGCTCGGGCAGAAGATTTCGATCACCTCCCGTAAAGCGCAGACTACGCGTCACCGTATTGTCGGGATCCATACCGAAGGGCCCTGGCAGGCGATTTACGTCGATACCCCCGGTCTGCACATGGAAGAAAAACGCGCGATTAACCGTCTGATGAACAAAGCGGCTAGCAGTTCCATTGGTGATGTTGAACTGGTGATCTTTGTGGTGGAAGGCACCCGCTGGACGCCGGATGACGAGATGGTGCTCAATAAACTGCGTGACGGTAAAGCGCCGGTGATTCTGGCCGTCAACAAAGTAGATAACGTGCAGGAAAAAGCCGATCTGCTGCCGCATTTGCAGTTCCTGGCAAGTCAGATGGACTTCCTCGACATCGTACCGATCTCTGCGGAAACCGGGATGAATGTCGACACCGTCGtctagagtcgaccggagaatggcgcgcgccctgtagcggcgcattaagcgcggcgggtgtggtggttacgcgcagcgtgaccgctacacttgccagcgccctagcgcccgctcctttcgctttcttcccttcctttctcgccacgttcgccggctttccccgtcaagctctaaatcgggggctccctttagggttccgatttagtgctttacggcacctcgaccccaaaaaacttgattagggtgatggttcacgtagtgggccatcgccctgatagacggtttttcgccctttgacgttggagtccacgttctttaatagtggactcttgttccaaactggaacaacactcaaccctatctcggtctattcttttgatttataagggattttgccgatttcggcctattggttaaaaaatgagctgatttaacaaaaatttaacgcgaattttaacaaaatattaacgcttacaatttaggtggcacttttcggggaaatgtgcgcggaacccctatttgtttatttttctaaatacattcaaatatgtatccgctcatnncangatcctttttaacccatcacatatacctgccgttcactattatttagtgaaatgagatattatgatattttctgaattgtgattaaaaaggcaactttatgcccatgcaacagaaactataaaaaatacagagaatgaaaagaaacagatagattttttagttctttaggcccgtagtctgcaaatccttttatgattttctatcaaacaaaagaggaaaatagaccagttgcaatccaaacgagagtctaatagaatgaggtcgaaaagtaaatcgcgcgggtttgttactgataaagcaggcaagacctaaaatgtgtaaagggcaaagtgtatactttggcgtcaccccttacatattttaggtctttttttattgtgcgtaactaacttgccatcttcaaacaggagggctggaagaagcagaccgctaacacagtacataaaaaaggagacatgaacgatgaacatcaaaaagtttgcaaaacaagcaacagtattaacctttactaccgcactgctggcaggaggcgcaactcaagcgtttgcgaaagaaacgaaccaaaagccatataaggaaacatacggcatttcccatattacacgccatgatatgctgcaaatccctgaacagcaaaaaaatgaaaaatatcaagttcctgagttcgattcgtccacaattaaaaatatctcttctgcaaaaggcctggacgtttgggacagctggccattacaaaacgctgacggcactgtcgcaaactatcacggctaccacatcgtctttgcattagccggagatcctaaaaatgcggatgacacatcgatttacatgttctatcaaaaagtcggcgaaacttctattgacagctggaaaaacgctggccgcgtctttaaagacagcgacaaattcgatgcaaatgattctatcctaaaagaccaaacacaagaatggtcaggttcagccacagttacatctgacggaaaaatccgtttattctacactgatttctccggtaaacattacggcaaacaaacactgacaactgcacaagttaacgtatcagcatcagacagctctttgaacatcaacggtgtagaggattataaatcaatctttgacggtgacggaaaaacgtatcaaaatgtacagcagttcatcgatgaaggcaactacagctcaggcgacaaccatacgctgagagatcctcactacgtagaagataaaggccacaaatacttagtatttgaagcaaacactggaactgaagatggctaccaaggcgaagaatctttatttaacaaagcatactatggcaaaagcacatcattcttccgtcaagaaagtcaaaaacttctgcaaagcgataaaaaacgcacggctgagttagcaaacggcgctctcggtatgattgagctaaacgatgattacacactgaaaaaagtgatgaaaccgctgattgcatctaacacagtaacagatgaaattgaacgcgcgaacgtctttaaaatgaacggcaaatggtacctgttcactgactcccgcggatcaaaaatgacgattgacggcattacgtctaacgatatttacatgcttggttatgtttctaattctttaactggcccatacaagccgctgaacaaaactggccttgtgttaaaaatggatcttgatcctaacgatgtaacctttacttactcacacttcgctgtacctcaagcgaaaggaaacaatgtcgtgattacaagctatatgacaaacagaggattctacgcagacaaacaatcaacgtttgcgccaagcttcctgctgaacatcaaaggcaagaaaacatctgttgtcaaagacagcatccttgagcaaggacaattaacagttaacaaataaaaacgcaaaagaaaatgccgatattgactaccggaagcagtgtgaccgtgtgcttctcaaatgcctgattcaggctgtctatgtgtgactgttgagctgtaacaagttgtctcaggtgttcaatttcatgttctagttgctttgttttactggtttcacctgttctgttaggtgttacatgctgttcatctgttacattgtcgatctgttcatggtgaacagctttaaatgcaccaaaaactcgtaaaagctctgatgtatctatcttttttacaccgttttcatctgtgcatatggacagttttccctttgatatctaacggtgaacagttgttctacttttgtttgttagtcttgatgcttcactgatagatacaagagccataagaacctcagatccttccgtatttagccagtatgttctctagtgtggttcgttgtttttgcgtgagccatgagaacgaaccattgagatcatgcttactttgcatgtcactcaaaaattttgcctcaaaactggtgagctgaatttttgcagttaaagcatcgtgtagtgtttttcttagtccgttacgtaggtaggaatctgatgtaatggttgttggtattttgtcaccattcatttttatctggttgttctcaagttcggttacgagatccatttgtctatctagttcaacttggaaaatcaacgtatcagtcgggcggcctcgcttatcaaccaccaatttcatattgctgtaagtgtttaaatctttacttattggtttcaaaacccattggttaagccttttaaactcatggtagttattttcaagcattaacatgaacttaaattcatcaaggctaatctctatatttgccttgtgagttttcttttgtgttagttcttttaataaccactcataaatcctcatagagtatttgttttcaaaagacttaacatgttccagattatattttatgaatttttttaactggaaaagataaggcaatatctcttcactaaaaactaattctaatttttcgcttgagaacttggcatagtttgtccactggaaaatctcaaagcctttaaccaaaggattcctgatttccacagttctcgtcatcagctctctggttgctttagctaatacaccataagcattttccctactgatgttcatcatctgaacgtattggttataagtgaacgataccgtccgttctttcctcgtagggttttcaatcgtggggttgagtagtgccacacagcataaaattagcttggtttcatgctccgttaagtcatagcgactaatcgctagttcatttgctttgaaaacaactaattcagacatacatctcaattggtctaggtgattttaatcactataccaattgagatgggctagtcaatgataattactagtccttttcctttgagttgtgggtatctgtaaattctgctagacctttgctggaaaacttgtaaattctgctagaccctctgtaaattccgctagacctttgtgtgttttttttgtttatattcaagtggttataatttatagaataaagaaagaataaaaaaagataaaaagaatagatcccagccctgtgtataactcactactttagtcagttccgcagtattacaaaaggatgtcgtaaacgctgtttgctcctctacaaaacagaccttaaaaccctaaaggcttaagtagcaccctcgcaagctcgggcaaatcgctgaatattccttctgtctccgaccatccggcacctgagtcgctgtctttttcgtgacattcagtccgctgcgctcacggctctggcagtgaatgggggtaaatggcactacaggcgccttttatggattcatgcaaggaaactacccataatacaagaaaagcccgtcacgggcttctcagggcgttttatggcgggtctgctatgtggtgctatctgactttttgctgttcagcagttcctgccctctgattttccagtctgaccacttcggattatcccgtgacaggtcattcagactggctaatgcacccagtaaggcagcggtatcatcaacaggcttacccgtcttactgtcnggatcgacgctctcccttatgcgactcctgcat

Plasmid annotation: nucleotide sequence 275-1069 is the kanamycin resistance gene; nucleotide sequence 1524-2023 and 2024-2533 are the homologous regions upstream and downstream of *rnc* gene respectively; nucleotide sequence 2558-3012 is the f1 bacteriophage origin of replication; nucleotide sequence 3116-4983 is the *sacB* gene expression cassette; nucleotide sequence 5376-6326 is the temperature-sensitive version of the RepA protein needed for replication with the pSC101 origin; nucleotide sequence 6374-6596 is the pSC101 origin.
