# Supplementary material for: A gut commensal bacterium promotes black soldier fly larval growth and development partly via modulation of intestinal protein metabolism
Source: mBio. 2023 Sep 14;14(5):e01174-23. doi: 10.1128/mbio.01174-23 (PMC10653789; doi:10.1128/mbio.01174-23)
Supplement: Text S3 — dsRNA target region of the genes screened in the study. [file mbio.01174-23-s0003.docx]

**Text S3** The dsRNA target region of the genes tested in the study.

**The dsRNA target region of *HiInR* (584 bp)**

TAACAGCGATTCGAGGCAACCATTTGTTCGAAGGATTCGCTCTTGTTATATTTGAAAATGCAAACATGGAGGAAATCGGTTTAACATCTCTAACAACAATTATTCGTGGTGGTGTTCGTATTGAGAAGAATCCTGCTCTTTGCTTTGTACATACAATCGATTGGAATCGAATCGCTATCAGCGCCAGTCCGGAAACGTTAGTTTTCAAGCACAATAAAGCAACGAACGAATGCCCGTCTTGTTGGCCTGGAAAAATGAAGGGCGAAGCGAACGGCGACTGCACTCCTGTGGCAAATAAGCGCTTTTGCTGGAACCAAACCCATTGCCAAAAAATTTGTCCTCCTGAATGTGGGAATCGAGCGTGCAATAGTCAGGGAAAGTGCTGCAATGAGACCTGTCTCGGGGATTGTTCCGCAGATGGGAATAAATGTACAGTATGTCGCCATTTGGTAGCTTATGGACGAAATGGCGAGAAAAGATGCGTGCAGGAATGCCCAGCAAATACGTACAAGCATATGAATCGGCGATGTGTGACGAAAGAAGAATGTATTGAAACTGCAAAGCCACTGCGGAGTAATTATGAG

**The dsRNA target region of *Hitrp1* (314 bp)**

ATGTTCCGTTTTGTAGTGTTAACCGCCTTGTTGGCCTGTGTATCAGGTGGACTCATCCCAGATTTGGATGGCCGCATTGTTGGAGGCAAAGCTACCAACATTGAAAGTTATCCTCATCAAGTTTCTTTGAGGAAGAGTGGTAGTCACATTTGTGGTGGTTCCATCTACAAACCAAATGTTGTTATCACCGCTGCTCACTGCACCCACGGACAATCGGCATCTTCCCTCACCGTCGTAGCCGGTACCAGTTCTCGTACTTCTGGAGGAGTTTCTCGTAAAGTATCCAGCATTCGTCAACATCCAAGTTACAGTGC

**The dsRNA target region of *Himtp1* (370 bp)**

AGCTAACTATGTTTACGTTACCCGTGAAAATTCTGGATGTTGGTCTTATGTTGGCATGTTGAAGGGACGTCAACAACTGAACTTGCAAGGAAACGGTTGTGTTTACCATGGAACTGCTATCCACGAATTCTTGCATGCTCTTGGTGTTCATCATCAACAAAGTGCTTCAGACCGTGACAACTATGTTACCATCCAATATGCTAATATCCAATCTGGAACTGAAGGAAACTTCGACAAATACTCCAGCAGTTACGTCACCGACTTCGGTATTGGTTACGATTATGGAAGTATCATGCACTACGATGCCTACGCCTTCAGCAAGAACGGTCAAAAGACCATTGTGACCAAGGACTCCAGTGCCACAATTGGA

**The dsRNA target region of *XM_038055439.1* (345 bp)**

CCAGCCAAGTTCGCATAAAAGATTGTAACCAGCCACCGTGTGCGGTAGTGAAAGGAACGACGGCCAACTTTCAGGTAGACTTCGAGGCAGCCTTCCCAATAAAAATTATGACAACAAAGGTTCGAGCTACTGTCCTGGGACTCACCGTCAACTATCCACTTGACGACGAGCAGGCGAATACATGCGCCCATTTGCTGTATGGGAGCTATTGTCCGCTAGATAGAGGCGAGGATATTACATACAATTTTGACTTTCCGGTCGGAAACTCATATCCGGAAATTGGAGTCAATGTTGAAGTTAGCTTGGAAGATGAATCAAAAAAGGTGGTGACATGCTTTAATGTTG

**The dsRNA target region of *XM_038069227.1* (321 bp)**

TGGCCCGATATTGGTTACAGTTTTGCCGTTGGTGGTGACGGTAACATCTACGAAGGTCGTGGCTGGAACGTTGAAGGAGCTCATACTCCAGGATACAATTCCCGAAGTGTTGGAATTTTGTTGATTGGTGATTTCAGGACCGCCTTGCCACCAGCAAACATGCTTACTGCTACCAAGAACTTCATTTCTCAAGGAGTTAACGAAGGAAGGATTTCAGGAAGCTATAAGTTGATTGGACACAGACAAGCTTATGCTACTGAATGCCCTGGAACCAGGTTGTACAATGAAATCAGGACCTGGCCCCGCTGGACCGCAAATCCT

**The dsRNA target region of *XM_038052062.1* (346 bp)**

ATGAAGGTCCTCGTTTGTTTGGTAGCTCTCATCGCCGTCGCTTCAGCAGGATATCTCGGCGGGTACGGTGGGGGATTCGGCGGAGGATACGGTGGCGGATACGGAGGATATGGTGGTTACGGCCATGGCGGCTATGGTGGCTTTGGTCATGGTGGTTATGGCGGCTACGGAGGACTCGGTGGATTAGGAGGAGGATACGGAGGGGGCATCGCCAAAGTCGTCAAAGTTGTAACTCCAGCTATTAGCATTGGCCATGGATTCGGCGGTGGATACGGAGGATACGGAGGAGGATACGGAGGTGGTTATGGCTTCGGAGGTGGCTATGGAGGTGGTTATGGAGGTGGCT

**The dsRNA target region of *XM_038059243.1* (410 bp)**

GCCATCGCAGTTATTGCCCTCCTCGTCCTCGTTTCCTGTGCTCAAGCCAGGATCTACACCCGTTGTGAAATGGCTCGTATTCTCTACCACGACTACGGTGTAACCAACTTGACCACTCTCGCCAACTGGATTTGTTTGATCCAACATGAATCAGGATTCAACGATCAAGCCGTCGGTGCCATCAACTACAACGGAACCCAAGATTTCGGTCTCTTCCAAATCAACAACCAATGGTGGTGTCAAGGAAACGTCTCCTCCTACAATAGCTGTGGAATCGCCTGTACCGCTCTTCTCGGAAACTTGCCAGCCTCCTGGAAGTGTGCCCAACTTGTCTACCAACAACAAGGTTTCAAGGCCTGGTACGGATGGCTCAACTACTGCAACGGAACTGCTCCAAGCGTTGCTGACTG

**The dsRNA target region of *XM_038060271.1* (389 bp)**

GGCCATCACTGCCGGTACCTCATACCGTACCAGCGGTGGAGTAACAAAGAAGGTCAGCAAGATCATTATCCATGAAAAATACAGCAGCAGCACTGTCGACAACGATGTCGCTATTTTGTTCCTTGAAGAAGGATTTGAACTTGGAGAATCCATTCAAACAATCAACCTCGCTTCAGCTGGATCTGTTGTTGAAGCTGGAGTCAAGGCAACCTGCAGTGGTTGGGGAGCTCTTAAAGAAGGTGGAGCCTCACCTTCTGTTCTCCAATTTGTTGATGTCTCCGTTGTAAACAATGCCGATTGTGGAGCAGCTTATGGCAAGGGTAGCATCACTGATGCTATGATGTGCGCTGGTGAAAAGCAAGGAGGAAAGGACGCTTGCCAAGGAGACT

**The dsRNA target region of *XM_038063869.1* (402 bp)**

GGTGGCGGTGATGGTGGATTCGGTGGTGGATACGGAGGTGGCCATGGTGGTGCTGTAATTAGCGGTGGATACGGTGGTGGTCATGGCGGTGGATACGGTGGTGGACACAATGCTGCAGCTAGTGTTGGCGCCGCACTTGGCGGTAGTCATAGTGCCGGAATCGTAGGCAATTATGCCGGAGGTCTTTCTGGTGGCCAATCTGCCGCATCTTTGGGACATGGAAGTTACGGTGGAGTCGAATTAGGCGGTGGACATGGTGGATATGATGGTGGTTACGAAGGAGGACATGGAGGATCTCTCGGTGGATATGAAGCCAGCTCCGGCGGACATGGTGGATCTTTGGGAGGATACGAAGGTGGTCATGGTGGATACGGAGGCTCAGCTGGAGGATCATATGGCGGA
