# Supplementary material for: A gut commensal bacterium promotes black soldier fly larval growth and development partly via modulation of intestinal protein metabolism
Source: mBio. 2023 Sep 14;14(5):e01174-23. doi: 10.1128/mbio.01174-23 (PMC10653789; doi:10.1128/mbio.01174-23)
Supplement: Supplemental figures — Figures S1 to S11. [file mbio.01174-23-s0004.docx]

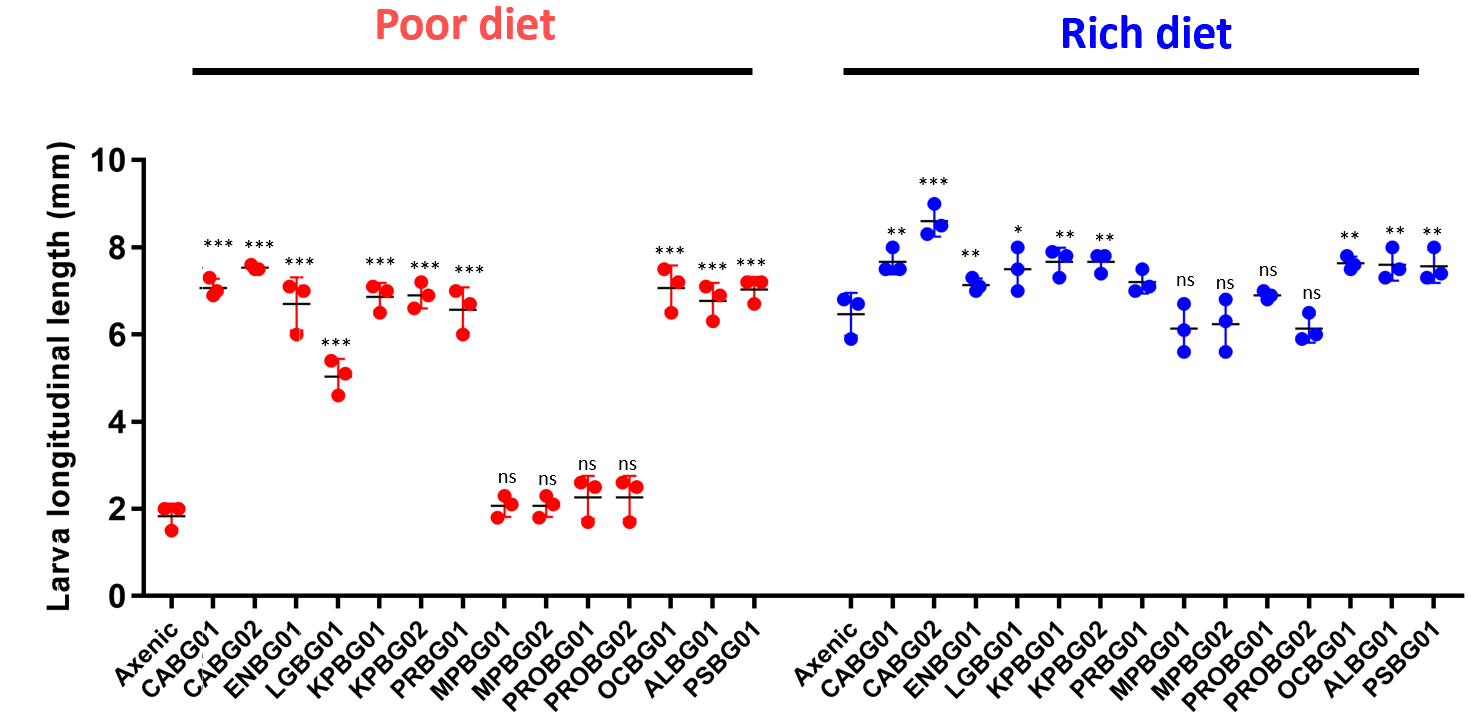
**FIG S1** The influence of intestinal isolates on BSFL growth. Larval longitudinal length at 6 dpi after the association of 1X living bacteria in both poor diet and rich diet with germ-free larvae. The control group consisted of the germ-free larvae with no bacterial association. Each treatment was replicated three times. The error bar represents Mean ± SD. Asterisks represent statistical significance compared to the control group. The statistical significance was determined by one-way ANOVA and Tukey’s multiple comparisons test with ^*^ *P* < 0.05, ^**^ *P* < 0.01, ^***^ *P* < 0.001, ns, not significant (*P* > 0.05).


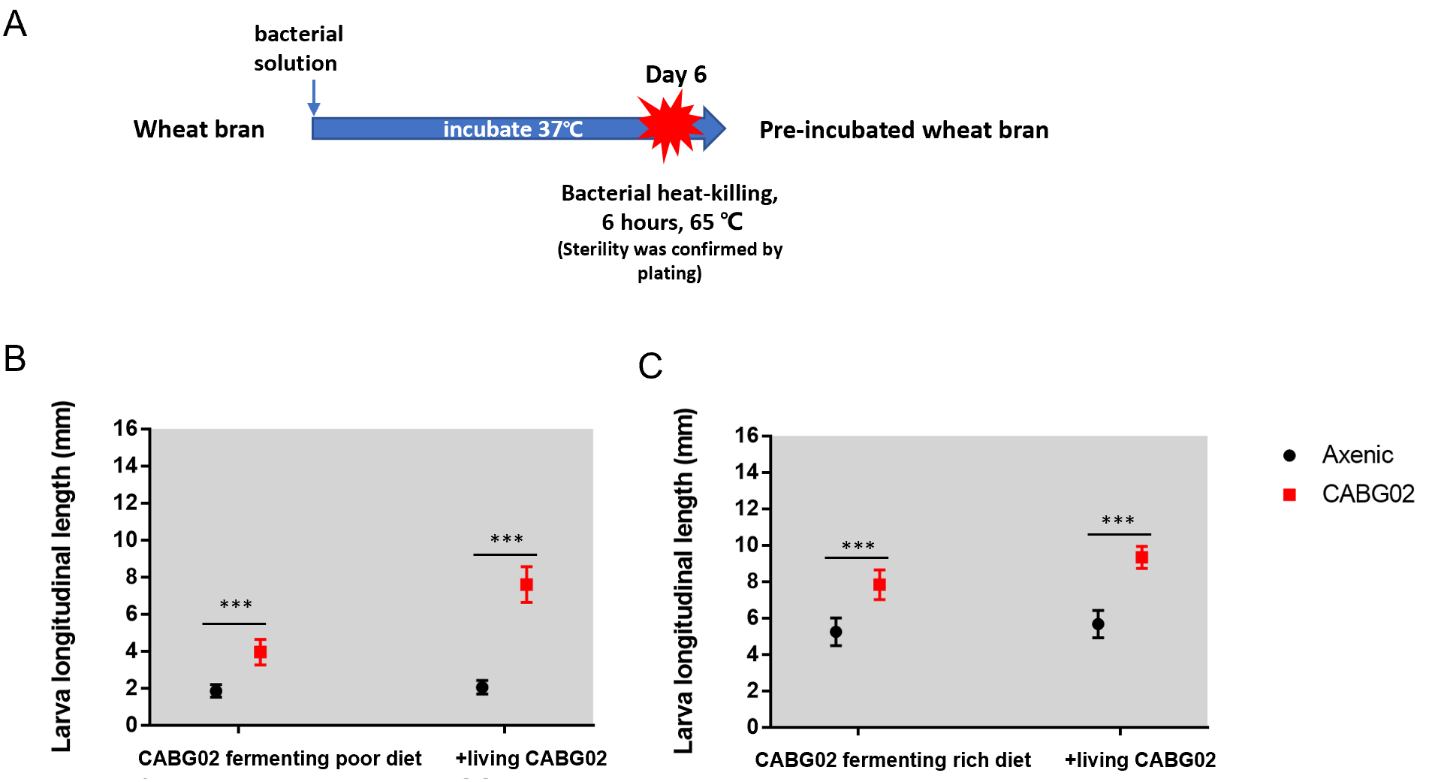
**FIG S2** The fermentation of substrates by CABG02 promoted larval growth. (A) Schematic diagram of the experiment. (B and C) Larval longitudinal length at 6 dpi in the fermented poor diet (B) and the rich diet (C) by 1X CABG02 (heat-killed before inoculating larvae) or the sterile diet where sterile larvae were associated with 1X living bacteria. Each treatment was replicated six times. The error bar represents Mean ± SD. Asterisks indicate statistical signiﬁcance between the two groups as determined by a two-tailed Student’s t-test with * *P* < 0.05, ** *P* < 0.01, and *** *P* < 0.001.


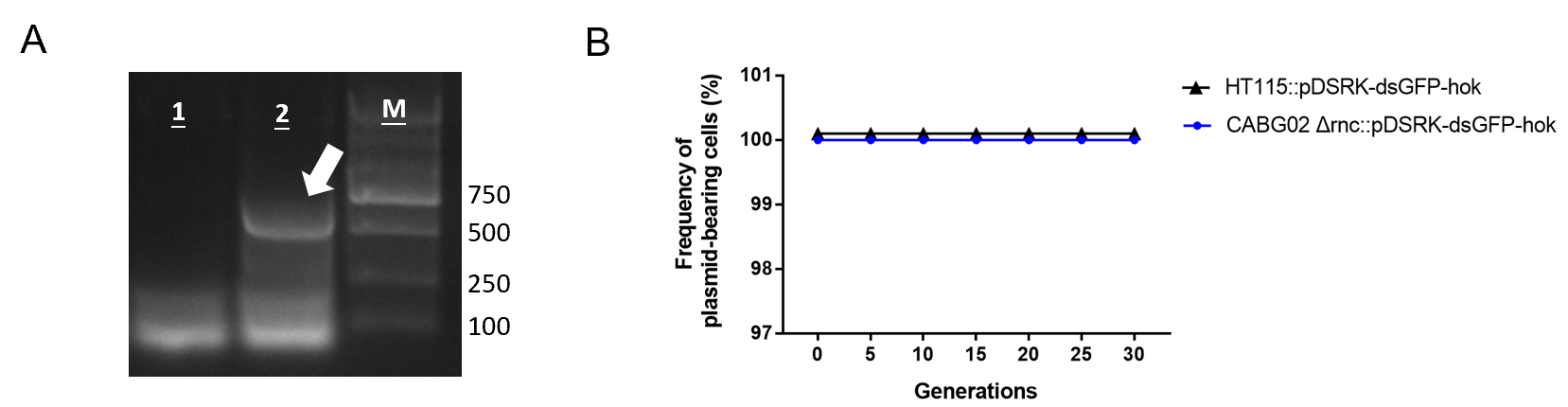


**FIG S3** The functionality of the pDSRK-dsGFP-hok dsRNA system in *E. coli* HT115. (A) 1% agarose gel electrophoresis of crude RNA extraction samples of HT115 (Lane 1) and HT115::pDSRK-dsGFP-hok (Lane 2). The arrow indicates the band of dsRNA. (B) The frequency of plasmid-bearing cells of bacterial populations of HT115::pDSRK-dsGFP-hok and CABG02 Δ*rnc*::pDSRK-dsGFP-hok after generations of cultivation. Each treatment was replicated three times.


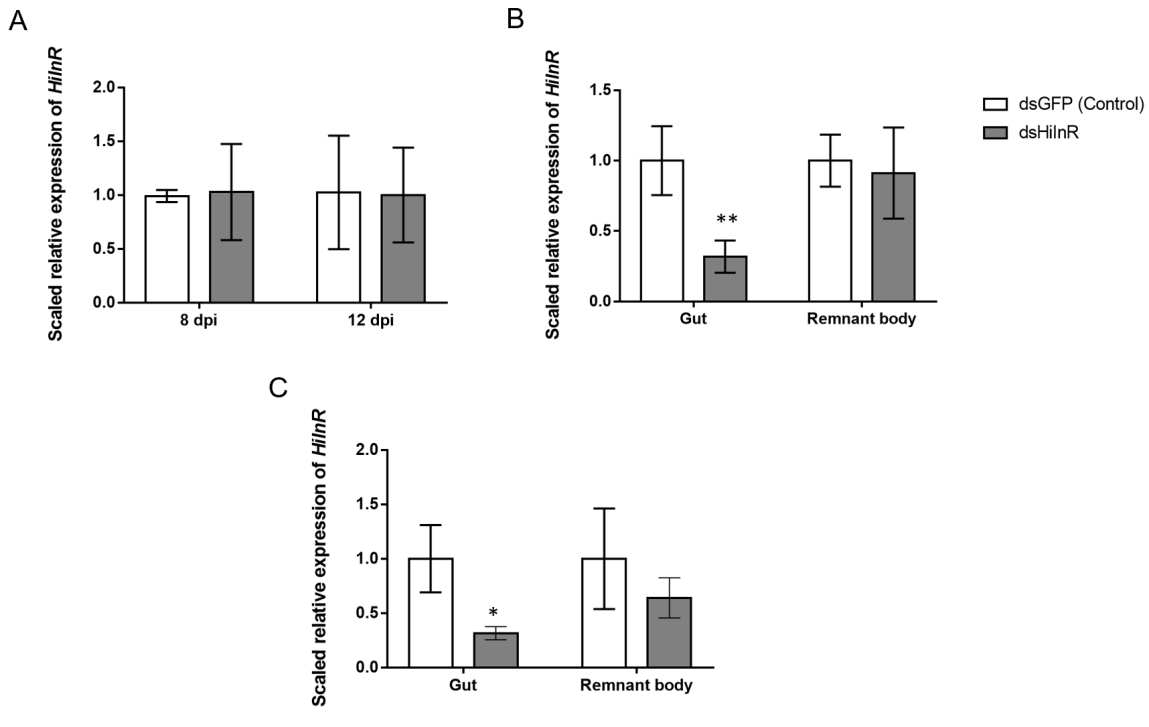


**FIG S4** *E. coli* HT115-mediated RNAi knocked down the expression of *HiInR* in BSFL intestines. (A) qRT-PCR examination on the expression of *HiInR* in the larvae whole body that were associated with 1X HT115::pDSRK-dsGFP-hok (control) and 1X HT115::pDSRK-dsHiInR-hok respectively at 8 dpi and 12 dpi. (B and C) qRT-PCR examination on the expression of *HiInR* in the guts and the remnant body of the larvae that were associated with 1X HT115::pDSRK-dsGFP-hok (control) and 1X HT115::pDSRK-dsHiInR-hok respectively at 6 dpi (B) and 12 dpi (C) respectively. Larval tissues from six individual larvae were grouped for each treatment. Each treatment was replicated four times. The error bar represents Mean±SD. Asterisks indicate statistical signiﬁcance compared to the control group as determined by a two-tailed Student’s t-test with ^*^ *P* < 0.05, ^**^ *P* < 0.01, and ^***^ *P* < 0.001.


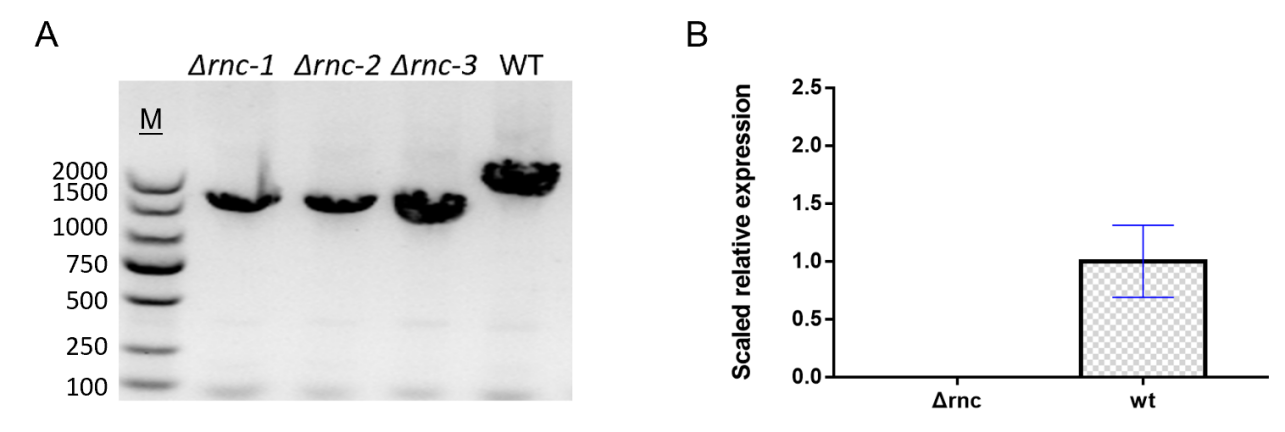
FIG S5 The construction of *C. amalonaticus* CABG02 Δ*rnc.* (A) PCR confirmation on the deletion of 642 bp in CABG02 Δ*rnc* strain. (B) qRT-PCR examination of *rnc* expression in CABG02 Δ*rnc* strain. Each treatment was replicated three times. The error bar represents Mean±SD.


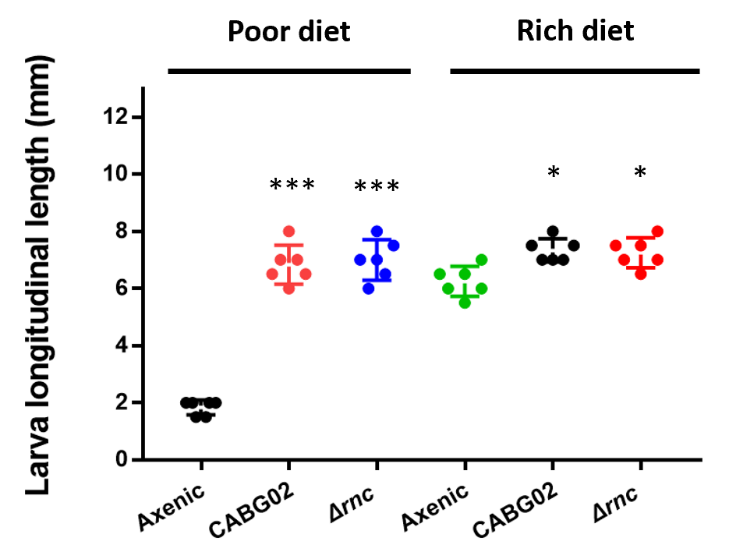
**FIG S6** The promoting effects of *C. amalonaticus* CABG02 Δ*rnc* on larval growth. Larval longitudinal length at 6 dpi of the axenic larvae (control) and the larvae associated with 1X CABG02 wildtype, 1X CABG02 Δ*rnc* in the poor diet and rich diet. Each treatment was replicated six times. The error bar represents Mean ± SD. Asterisks indicate statistical signiﬁcance compared to the control group as determined by a two-tailed Student’s t-test with ^*^ *P* < 0.05, ^**^ *P* < 0.01, and ^***^ *P* < 0.001.


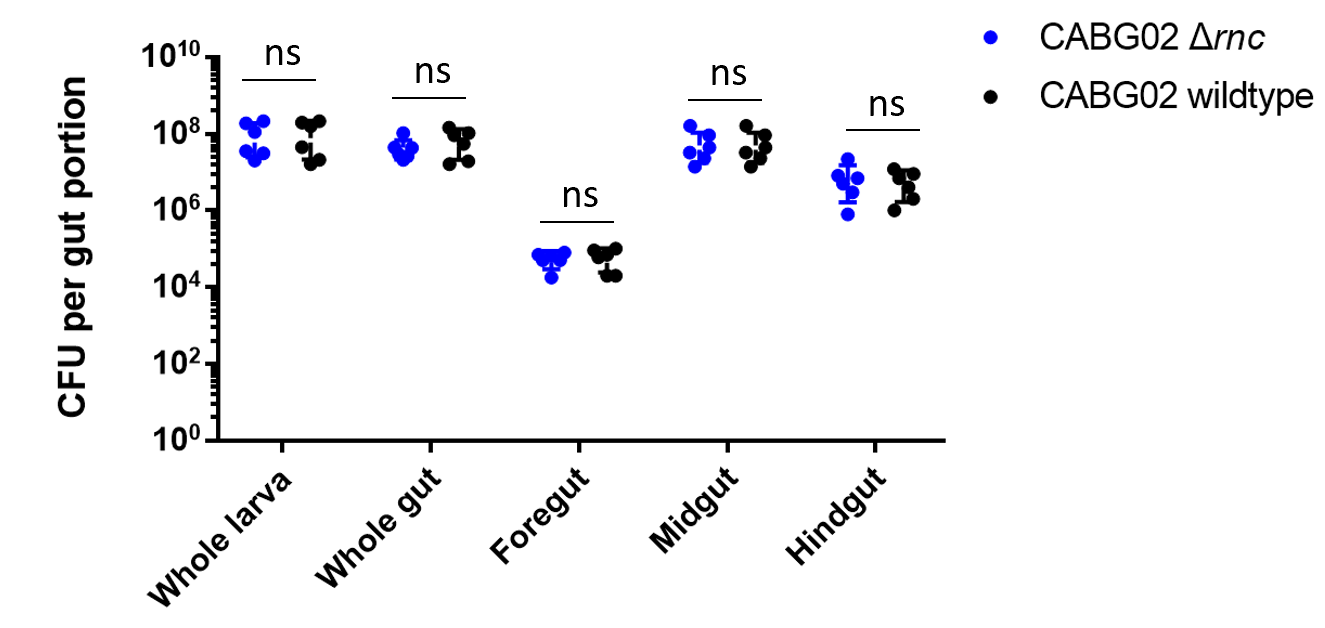


**FIG S7** Distribution of CABG02 Δ*rnc* and the wildtype strain in larval gut portions of the larvae associated with the respective strain (1X). Larval tissues from five individual larvae were grouped for each treatment. Each treatment was replicated six times. The error bar represents Mean ± SD. Asterisks above horizontal bars represent statistical significance between the groups. The statistical significance was determined by a two-tailed Student’s t-test with * *P* < 0.05, ** *P* < 0.01, *** *P* < 0.001, ns, not significant (P > 0.05).


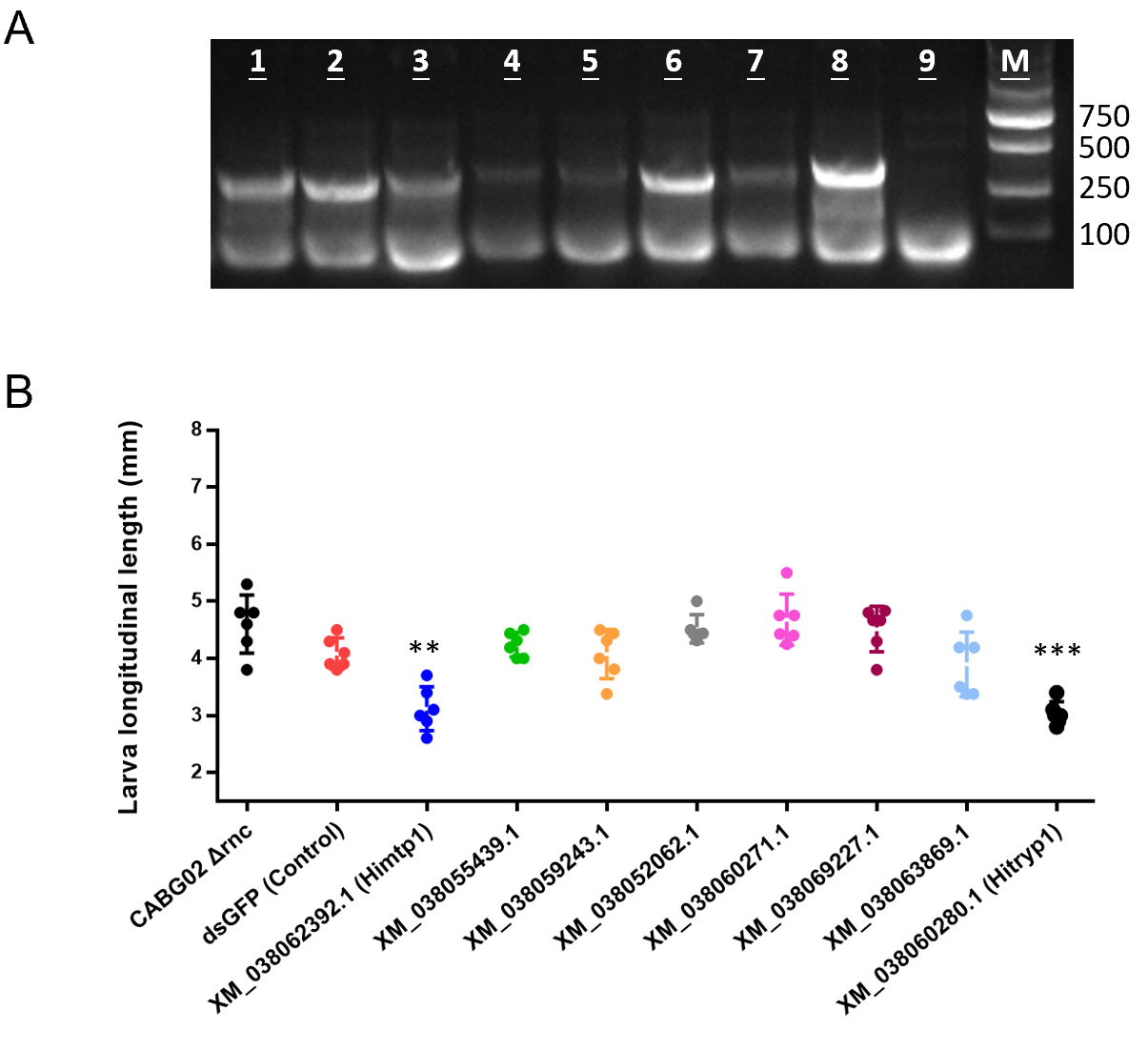
**FIG S8** Screen of the candidate DEGs involved in the interaction by the symbiont-mediated RNAi. (A) 1% agarose gel electrophoresis of crude RNA extraction samples of CABG02 Δ*rnc* transformed with a series of dsRNA-producing plasmids targeting XM_038062392.1 (Lane 1), XM_038060280.1 (Lane 2), XM_038055439.1 (Lane 3), XM_038059243.1 (Lane 4), XM_038052062.1 (Lane 5), XM_038060271.1 (Lane 6), XM_038069227.1 (Lane 7), XM_038063869.1 (Lane 8) and CABG02 Δ*rnc* with no plasmid (Lane 9). (B) Larval longitudinal length at 4 dpi after the association of 1X CABG02 Δ*rnc*, 1X CABG02 Δ*rnc*::pDSRK-dsGFP-hok (control), and 1X CABG02 Δ*rnc* transformed with a series of dsRNA-producing plasmids targeting the DEGs. Each treatment was replicated six times. The error bar represents Mean ± SD. Asterisks indicate statistical signiﬁcance compared to the control group as determined by one-way ANOVA and Tukey’s multiple comparisons test with * *P* < 0.05, ** *P* < 0.01, and *** *P* < 0.001.

**FIG S9** A multiple alignment of coding sequences of *Hitryp1* and the eight potential duplicates in BSF genome. The dsRNA targeting region ranges from 1 to 315 nucleotide.


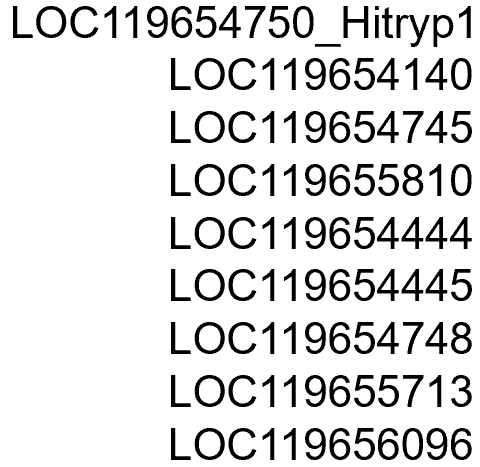

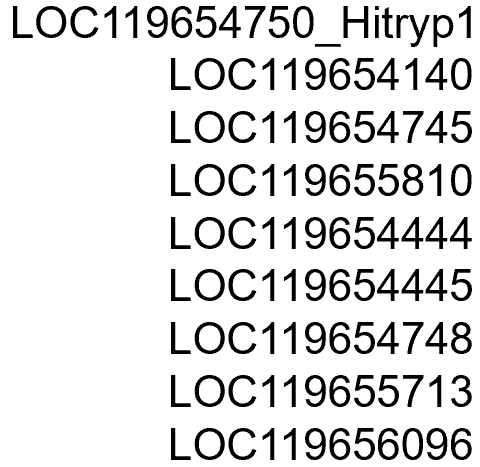

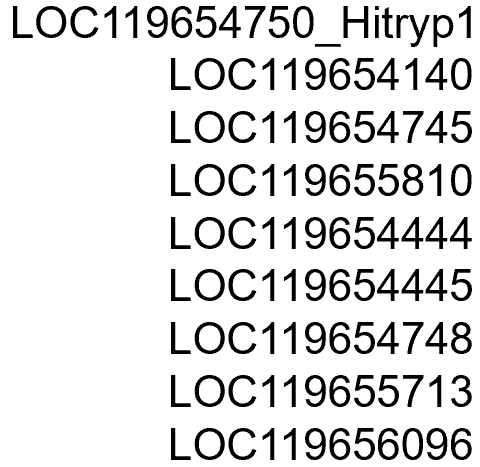

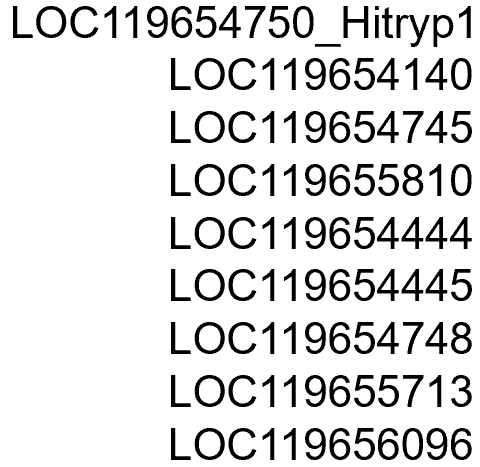

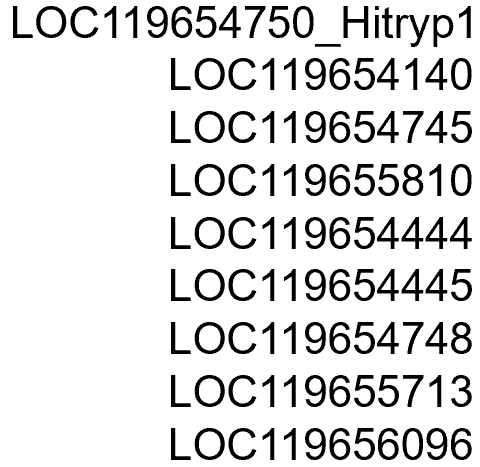

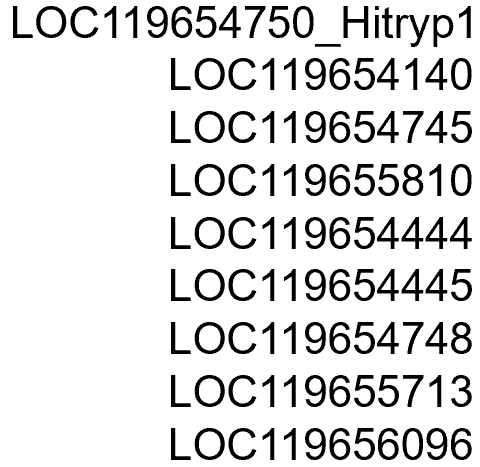

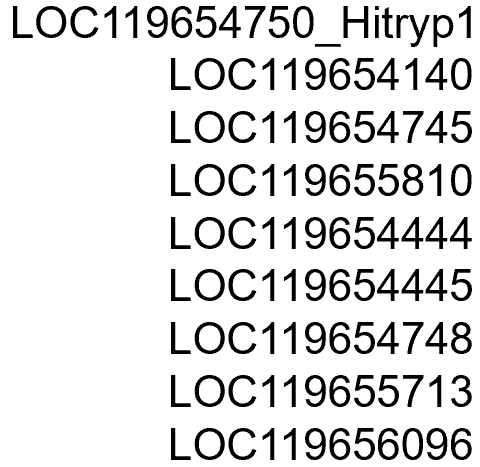

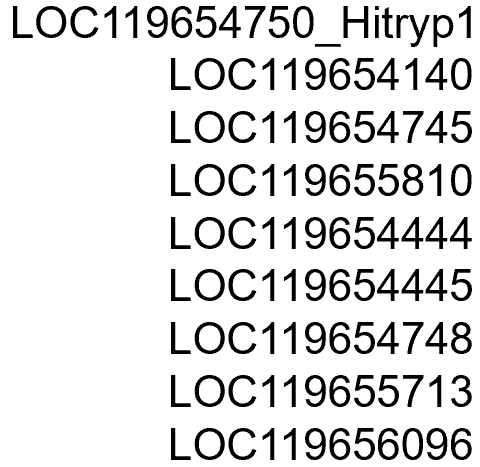

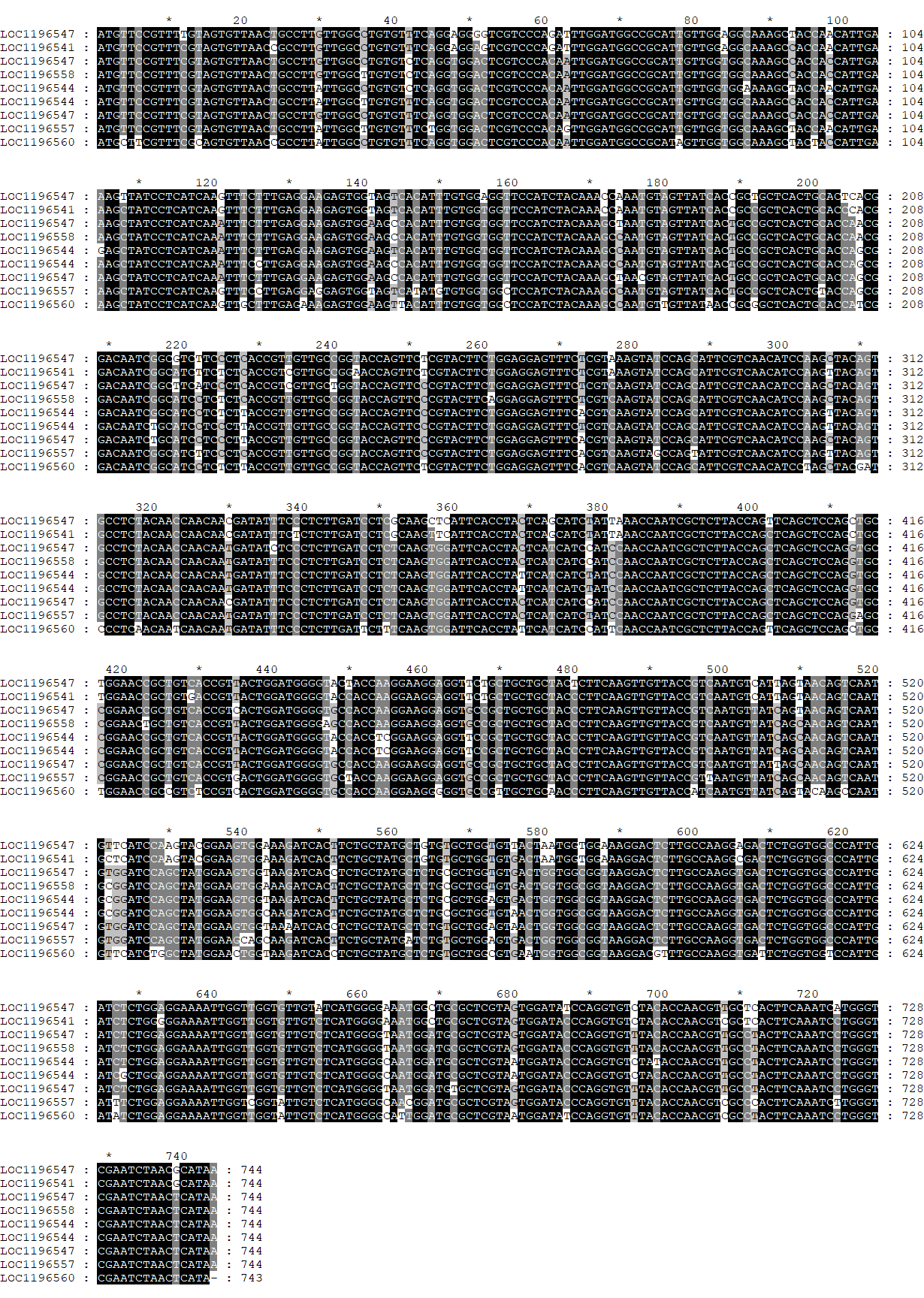


**FIG S10** A multiple alignment of coding sequences of *Himtp1* and the three potential duplicates in BSF genome. The dsRNA targeting region ranges from 293 to 663 nucleotide.


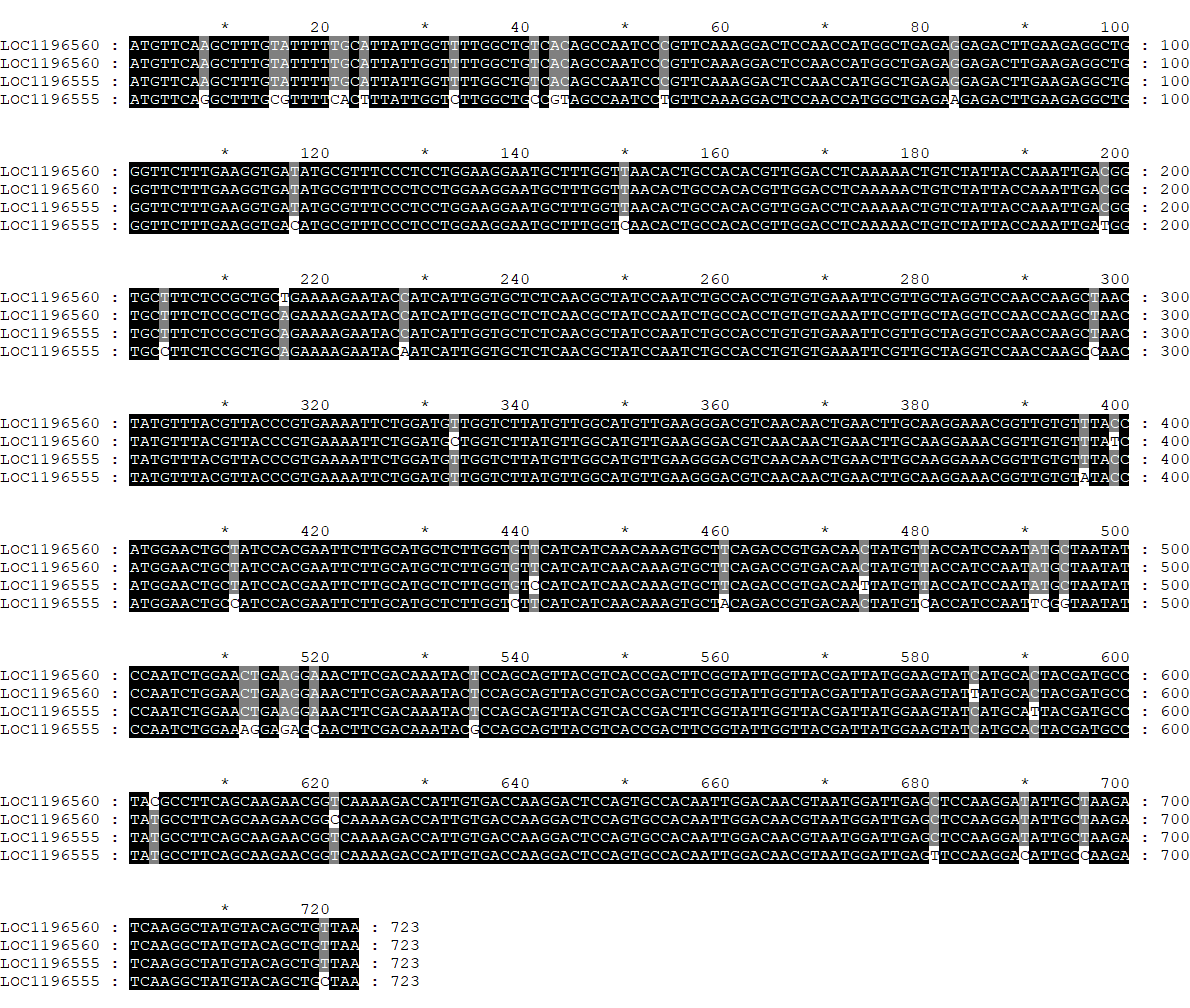

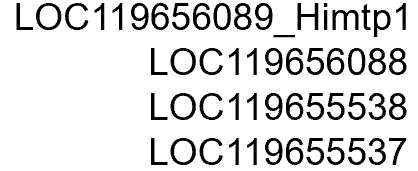

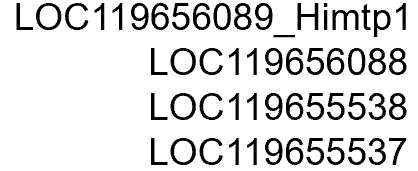

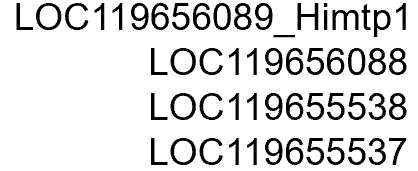

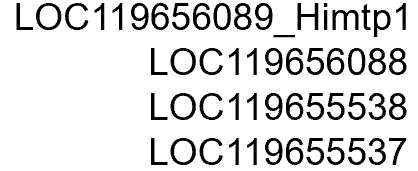

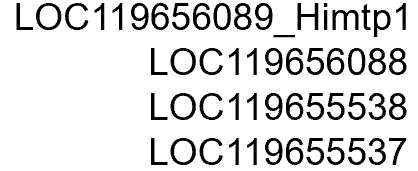

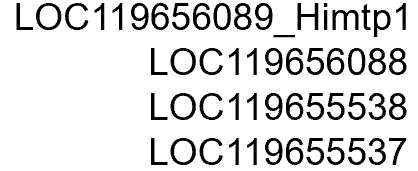

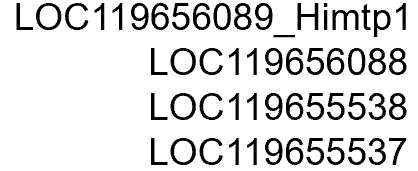

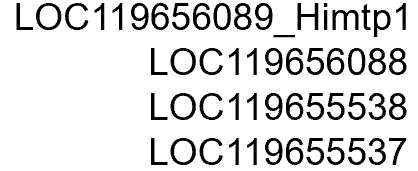


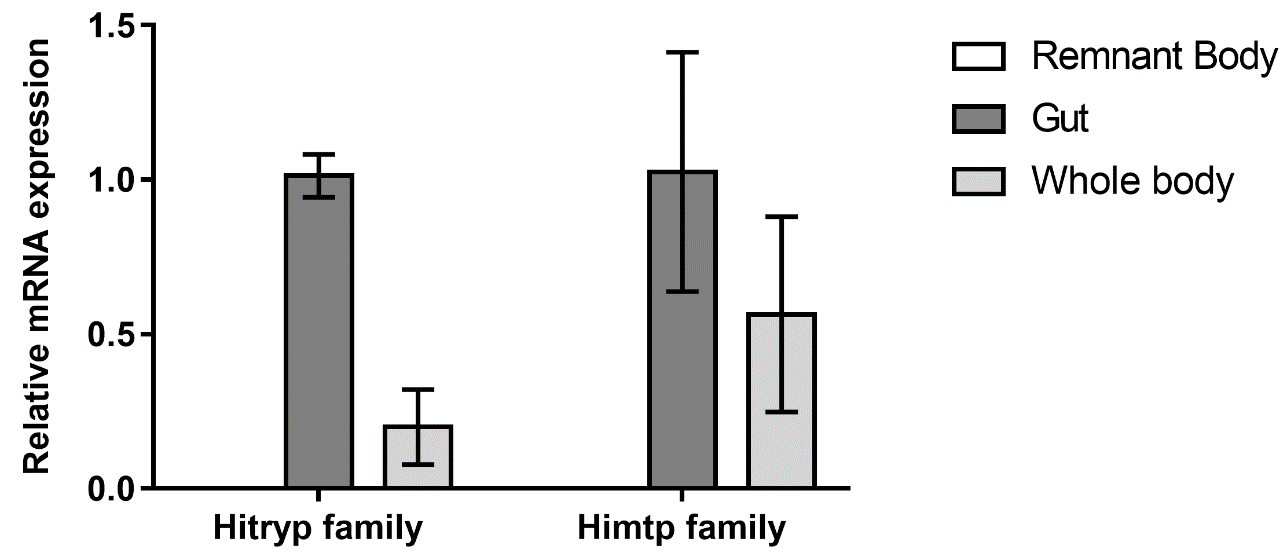
**FIG S11** Hitryp and Himtp family were expressed mainly in the BSF larval guts. qRT-PCR examination on the expression of *Hitrp1* and *Himtp1* in larval whole body, the intestine and the remnant body of larvae associated with 1X CABG02 Δ*rnc* at 8 dpi. Larval tissues from six individual larvae were grouped for each treatment. Each treatment was replicated four times. The error bar represents Mean ± SD.
