# Supplementary material for: A gut commensal bacterium promotes black soldier fly larval growth and development partly via modulation of intestinal protein metabolism
Source: mBio. 2023 Sep 14;14(5):e01174-23. doi: 10.1128/mbio.01174-23 (PMC10653789; doi:10.1128/mbio.01174-23)
Supplement: Legends — to the supplemental tables and texts. [file mbio.01174-23-s0005.docx]

**Legends to the supplemental tables and texts**

**Table S1** 16S rRNA gene sequence and classification of the isolated intestinal bacterial strains.

**Table S2** Gene expression (RNA-Seq) of axenic larvae versus larvae mono-associated with CABG02 at 2 dpi. Columns are as follows: Gene ID; read counts for each sample; LogFC, log2(fold change); pvalue; FDR-corrected P value.

**Table S3** The list of the DEGs in the Toll and Imd signaling pathway from the RNA-seq analysis.

**Table S4** The list of primers used in the study.

**Text S1** Supplemental methods.

**Text S2** The sequence and annotation of plasmids generated in the study.

**Text S3** The dsRNA target region of the genes tested in the study.
