# Supplementary material for: A gut commensal bacterium promotes black soldier fly larval growth and development partly via modulation of intestinal protein metabolism
Source: mBio. 2023 Sep 14;14(5):e01174-23. doi: 10.1128/mbio.01174-23 (PMC10653789; doi:10.1128/mbio.01174-23)
Supplement: Table S3 — DEGs in the Toll and Imd signaling pathway from the RNA-seq analysis. [file mbio.01174-23-s0008.docx]

**Table S3** The list of the DEGs in the Toll and Imd signaling pathway from the RNA-seq analysis.

| Gene ID | Annotation | 2dpi_Log2(fc) | Expression pattern compared to the Control |
| --- | --- | --- | --- |
| XP_037923272.1 | peptidoglycan-recognition protein SB1-like | -2.43 | Down |
| XP_037923463.1 | peptidoglycan-recognition protein SB1-like | -4.09 | Down |
| XP_037923643.1 | peptidoglycan-recognition protein SB1-like | -3.97 | Down |
| XP_037925150.1 | peptidoglycan-recognition protein LB-like | 2.81 | Up |
| XP_037925152.1 | peptidoglycan-recognition protein LB-like | 1.03 | Up |
| XP_037923272.1 | peptidoglycan-recognition protein LB-like | -2.32 | Down |
| XP_037923635.1 | peptidoglycan-recognition protein LB-like | 2.60 | Up |
| XP_037925153.1 | peptidoglycan-recognition protein LB-like | 2.01 | Up |
| XP_037925157.1 | peptidoglycan-recognition protein LB-like | 2.00 | Up |
| XP_037925156.1 | peptidoglycan-recognition protein LB-like | 2.32 | Up |
| XP_037925155.1 | peptidoglycan-recognition protein LB-like | 3.71 | Up |
| XP_037912269.1 | dual oxidase | 1.06 | Up |
