# Supplementary material for: A gut commensal bacterium promotes black soldier fly larval growth and development partly via modulation of intestinal protein metabolism
Source: mBio. 2023 Sep 14;14(5):e01174-23. doi: 10.1128/mbio.01174-23 (PMC10653789; doi:10.1128/mbio.01174-23)
Supplement: Table S4 — The primers used in the study. [file mbio.01174-23-s0009.docx]

**Table S4** The list of primers used in the study.

| Primer | Sequence（5’-3’） |
| --- | --- |
| 16S_qRT-PCR F/R | CGGTGAATACGTTCYCGG |
|  | GGWTACCTTGTTACGACTT |
| 26F / 1492R | CGGTGAATACGTTCYCGG |
|  | GGWTACCTTGTTACGACTT |
| pdsHitrp F/R | GAGCTCTCATCGTGAATACTATGTTCCGTTTTGTAGTGTT |
|  | GATATCCGTGACTAGACTGTGCACTGTAACTTGGATGTTG |
| pdsHimtp1 F/R | gagctcTCATCGTGAATACTAGCTAACTATGTTTACGTTACC |
|  | ttGATATCCGTGACTAGACTGTTCCAATTGTGGCACTGGAGT |
| pdsXM_038069227.1 F/R | ggtataatcacatagtactgttTGGCCCGATATTGGTTACAG |
|  | tataatcacatagtactgttAGGATTTGCGGTCCAGCGGG |
| pdsXM_038055439.1 F/R | gtataatcacatagtactgttCCAGCCAAGTTCGCATAAAA |
|  | tataatcacatagtactgttCAACATTAAAGCATGTCACC |
| pdsXM_038052062.1 F/R | tataatcacatagtactgttATGAAGGTCCTCGTTTGTTT |
|  | tataatcacatagtactgttAGCCACCTCCATAACCACCT |
| pdsXM_038059243.1 F/R | tataatcacatagtactgttGCCATCGCAGTTATTGCCCT |
|  | ggtataatcacatagtactgttCAGTCAGCAACGCTTGGAGC |
| pdsXM_038060271.1 F/R | tataatcacatagtactgttGGCCATCACTGCCGGTACCT |
|  | ggtataatcacatagtactgttAGTCTCCTTGGCAAGCGTCC |
| pdsXM_038063869.1 F/R | tataatcacatagtactgttGGTGGCGGTGATGGTGGATT |
|  | ggtataatcacatagtactgttTCCGCCATATGATCCTCCAG |
| EGFP_cp25 F/R | ataatcacatagtactgttaattcattaaagaggagaaaggtaccatggtgagcaagggcgag |
|  | tataatcacatagtactgttttacttgtacagctcgtcca |
| pBBR_cp25 F/R | tgcgcaactgttgggaagggctttggcagtttattcttga |
|  | tgtcggcagaatgcttaatgaattacaacagttttgacaaatgctctttccct |
| Hi_actin F/R | CGTAGGAGACGAAGCACAAA |
|  | GGTGCCAGATCTTCTCCATATC |
| Hitryp1_qRT-PCR F/R | TCGCAAGTTCATTCACCTAC |
|  | ACTTCCGTATTTGGATGAAC |
| Himtp1_qRT-PCR F/R | ACGTTGGACCTCCAAAACTG |
|  | TGGACCTAGCAACGAATTTC |
| pUCKan F/R | tatccgcctccatccagtcttagactgggcggttttatgg |
|  | accctgataaatgcttcaataaatctcgtgatggcaggtt |
| pMThoksok F/R | gcgtttcggtgatgacggtgactagtcttggactcctgtt |
|  | ttaagccagccccgacacccatttaaatacaacatcagca |
| pDSRKREV F/R | tgctgatgttgtatttaaatgggtgtcggggctggcttaa |
|  | aacaggagtccaagactagtcaccgtcatcaccgaaacgc |
| pdsGFP F/R | tataatcacatagtactgttccacaagttcagcgtgtccg |
|  | ggtataatcacatagtactgttggctgttgtagttgtactcc |
| pdsInR F/R | gagctcTCATCGTGAATACTTAACAGCGATTCGAGGCAAC |
|  | GATATCCGTGACTAGACTGTCTCATAATTACTCCGCAGTG |
| pKOVKan F/R | gccctgggccaacttttggctagactgggcggttttatggaca |
|  | tggtgaaactcacccagggaaaatctcgtgatggcaggtt |
| pKOV_KO_rnc_up F/R | ttaatcggtacccggggatcAGCCGCTGGCGACCTGGATT |
|  | GCTCATTCCAGCTCCAGTTTATTAATTACGATGGGGTTCA |
| pKOV_KO_rnc_down F/R | TGAACCCCATCGTAATTAATAAACTGGAGCTGGAATGAGC |
|  | attctccggtcgactctagaCGACGGTGTCGACATTCATC |
| pKOVKOrnc_confirm F/R | TTACGCGCTTCAATACCGAT |
|  | TCCCGCTGAGTGAAACGAAA |
| HiInR_qRT-PCR F/R | AAGCTCCTTTCCAGAACGTG |
|  | TACTGGGTATATGGTTTCAG |
| rnc_qRT-PCR F/R | TAATCGGCTTCAACGGAAGC |
|  | ATACGGCTCATATCACCCTC |
